# A Subvoxel Correction of Boiling‐Induced Susceptibility Artifacts in Magnetic Resonance Thermometry and Dosimetry for Monitoring Microwave Thermoablation: A Feasibility Study in a Swine Model

**DOI:** 10.1002/mrm.70129

**Published:** 2025-10-16

**Authors:** Eber Dantas, Rebecca Lafont, Pierre Bour, Thibaut Faller, Bruno Quesson, Helcio R B Orlande, Valéry Ozenne

**Affiliations:** ^1^ Politécnica/COPPE, Department of Mechanical Engineering Federal University of Rio de Janeiro—UFRJ Rio de Janeiro Brazil; ^2^ CNRS CRMSB, UMR 5536 IHU Liryc Université de Bordeaux Bordeaux France; ^3^ Certis Therapeutics Pessac France

**Keywords:** boiling artifact, microwave ablation, MRI, susceptibility bubbles, thermal dose, thermometry

## Abstract

**Purpose:**

Real‐time monitoring of microwave liver ablation (MWA) using MRI thermometry can be hindered by boiling‐induced susceptibility artifacts. These artifacts cause large temperature measurement errors that prevent accurate lesion size prediction. This study proposes a correction methodology based on removing the contribution of this susceptibility artifact using subvoxel sources of susceptibility.

**Methods:**

In vivo microwave ablations (*N* = 23) were performed on seven pig livers and the temperature was monitored using the proton resonance frequency shift (PRFS) method. The boiling‐induced artifacts were observed in 11 ablation cases. To validate the proposed methodology, the thermal dose was computed from the corrected temperature maps, and the resulting lesion estimates were compared in three dimensions with ground‐truth lesion segmentations derived from post‐ablation T1w images. A numerical simulation was also conducted to emphasize the need for a finer spatial discretization.

**Results:**

After correction, a better agreement was noticed between thermal dose prediction and lesion size. Over the 11 cases observed with boiling‐induced susceptibility artifacts, the median volumetric Dice, Total Overlap, and False Negative rates changed by 4.8%, 6.2%, and −11.4%.

**Conclusion:**

Comparison with T1w imaging showed improvements in prediction of lesion volume. The proposed methodology takes into account partial volume effects and the contribution of adjacent slices. It was able to simulate a diverse range of deformed dipole‐like artifacts observed in experimental data.

## 1 Introduction

Thermoablation is one of the main treatments against early‐stage hepatocellular carcinoma (HCC) [1, 2, 3], by thermally destroying pathological tissues with local energy delivery. The target volume (ablation zone) must cover the tumor tissue, while minimizing the damage to healthy surroundings. Thermoablation clinical procedures contain inherent variabilities concerning the ablation probe position, patient anatomy and perfusion effects, leading to uncertainties in the obtained lesion size. Thus, thermoablation treatments are associated with a 6%–12% local recurrence due to incomplete lesion coverage [4, 5].

A promising improvement on this treatment is the use of real‐time temperature monitoring via MRI [6, 7, 8], which enables visualization of the heating zone during ablation [9, 10], and the quantitative evaluation of the thermal dose (TD) [11, 12, 13]. The standard image‐guided single‐probe treatment for HCC assumes a maximum tumor size of 3 cm [14]. As the volume required for the ablation zone is significant, its coverage should be monitored in 3D [13, 15].

Real‐time MR thermometry is mainly based on the proton resonance frequency shift (PRFS) effect [16, 17, 18]. Fast image acquisition obtained with gradient echo (GRE) is used to perform phase mapping through time, and its relative change is transformed to relative temperature.

PRFS thermometry is highly impacted by susceptibility artifacts [19, 20], for example, due to background air movement (mainly in hyperthermia [21, 22, 23]). This work focuses on boiling‐induced bubble formation that generates dipole‐shaped susceptibility artifacts, characterized by a localized decrease in the magnitude signal and large temperature errors [24].

Recently, these artifacts were observed in 44% of cases in laser interstitial thermal therapy (LITT) performed on brain tumors [25, 26]. In silico investigations [27] confirmed that the observed artifacts were induced by bubbles rather than hemorrhage. A recent preclinical study [28] investigated this problem for liver microwave liver ablation (MWA), where high target temperatures are required due to high perfusion in liver. Nevertheless, the susceptibility estimation and temperature correction proposed in their work [28] were optimized retrospectively, using the true lesion size estimated from T1w postprocedural images, therefore it cannot be applied during treatment.

Despite renewed interest in susceptibility artifact corrections [21, 22, 23], current methods do not meet the technical challenges imposed by thermoablation, which are more complex than in hyperthermia. First, the location of the artifact is near the voxels where the temperature measurements are used for numerical computations. Secondly, in hyperthermia, the cavities with moving background air can be inferred from the anatomy based on magnitude images [22], while thermoablation introduces uncertainties regarding the exact positioning of individual bubbles, which might be smaller than the voxels. Besides, currently available correction approaches are also limited to specific continuous shapes, with a single value of susceptibility for simulation of the bubbles [21, 22, 23, 28], or a specific single‐parameter distribution [24].

This work proposes a correction for temperature images corrupted by boiling‐induced susceptibility artifacts. It consists in generating a susceptibility distribution that reproduces the main stable component of the artifact through time. Susceptibility values are obtained by solving an inverse problem that uses the dipole model of the susceptibility effect. A specific methodology was developed that uses a finer resolution (subvoxels) for the simulation of the susceptibility bubbles before generating the artifact in the required data resolution (voxels) and applying the correction. The methodology takes into account: (i) partial volume effects; (ii) adjacent slices contribution; and (iii) the fact that dipole models induce deviations that grow without bounds close to the dipole position. A numerical study is presented first to illustrate the importance of spatial discretization and multi‐slice simulation. The correction is then applied to 11 in vivo multi‐slice MRI cases of MWA performed in pig liver. The results are qualitatively evaluated using the temperature images from multiple slices, time series, TD maps and lesion sizes. Finally, the TD‐estimated lesions are compared in 3D to the lesions observed on post‐contrast T1w images.

## 2 Theory

The PRFS effect is summarized as [17, 29]:

(1) $$\Delta T=\frac{\Delta \Phi }{\alpha B_{0}\gamma \mathrm{TE}},$$

where ΔΦ is the phase change between the current and reference time, ΔT the corresponding local temperature variation (°C), γ/(2π)= 42.58 MHz/*T* the gyromagnetic ratio, α= −0.0094 ppm/°C the PRFS coefficient, and TE the echo time (seconds). Perturbations in the magnetic susceptibility of the medium impact the phase, which may be misinterpreted as a temperature variation, as explained below.

Considering the $B_{0}$ direction, a linear isotropic medium, neglecting second‐order contributions [30], and considering that the tissue susceptibility is small, the screening of the hydrogen nuclei [31] establishes:

(2) $$\begin{array}{c} B_{\mathrm{nuc}}=\left(1-\sigma -\frac{2}{3}\chi \right)\left(1+\chi \right)\left(B_{0}+\mu_{0}H_{\text{susc}}\right), \end{array}$$

where $B_{\mathrm{nuc}}$ is the nucleus local field, σ the proton–electron screening constant, χ the susceptibility, $\mu_{0}$ the vacuum permeability (N/A^2^), and $H_{\text{susc}}$ the auxiliary field related to χ. After further linearizations in Maxwell equations [30, 32, 33]:

(3) $$\begin{array}{c} \frac{B_{\mathrm{nuc}}-B_{0}}{B_{0}}=-\sigma +d⊗\chi , \end{array}$$

where d is the dipole kernel (volume^−1^), and ⊗ the spatial convolution. The left‐hand side of Equation (3) is the Relative Difference Field (RDF): the measurable part of the equation.

For the PRFS measurement, values at a reference time are subtracted from Equation (3):

(4) $$\begin{array}{c} \text{ΔRDF}=-\Delta \sigma +d⊗\Delta \chi . \end{array}$$

After introducing the PRFS, Δσ=−αΔT, multiplication by $B_{0}\gamma \mathrm{TE}$ reveals how the measured phase is affected:

(5) $$\begin{array}{c} \Delta \phi_{\text{meas}}=\left(\alpha B_{0}\gamma \mathrm{TE}\right)\Delta T_{\text{true}}+\left(B_{0}\gamma \mathrm{TE}\right)\left(d⊗\Delta \chi \right), \end{array}$$

where the first term on the right‐hand side corresponds to the true temperature variation, $\Delta T_{\text{true}}$. Therefore, when generating temperature images, direct use of the PRFS relation for $\Delta \phi_{\text{meas}}$ will provide a corrupted temperature variation $\Delta T_{\text{meas}}$. If a choice of Δχ can simulate the artifact, it can then be subtracted out to estimate $\Delta T_{\text{true}}$:

(6) $$\begin{array}{c} \Delta T_{\text{true}}=\Delta T_{\text{meas}}-\frac{1}{\alpha }\left(d⊗\Delta \chi \right)\;. \end{array}$$

The success of this strategy depends on the model used for simulation. The dipole kernel is [30, 33]:

(7) $$\begin{array}{c} d\left(x\right)=\frac{1}{4\pi }\frac{3{\left(x\cdot n\right)}^{2}-{\left|x\right|}^{2}{\left|n\right|}^{2}}{{\left|x\right|}^{5}}, \end{array}$$

where x≠0 is the spatial position, and n is the normalized $B_{0}$ direction (d=0 for x=0) [33, 34].

## 3 Methods

### 3.1 Correction Methodology

We consider two fundamental assumptions based on experimental observations:

1. Bubble formation/establishment is considered instantaneous (few seconds) in comparison to heating duration (7.5 min).
2. The bubble‐induced susceptibility artifact is assumed temporally constant after bubble establishment.

The representative situation (Figure S1) is that ablation starts and proceeds without boiling for a period of time until an artifact appears. The spatial increase of the signal‐void in the magnitude image is accompanied by unrealistically negative temperatures variations along one of the directions. Although the artifact is not perfectly constant through time, it can be noticed that some stability is reached. The instantaneous time of bubble formation was manually determined (white lines in Figure S1C,E) by examining the temporal evolution of the spatial profiles of magnitude and temperature.

Our correction methodology consists in estimating the subvoxel susceptibility distribution that reproduces this stable state of the boiling‐induced artifact, in order to simulate the temperature errors caused by the artifact, and then subtract them from the original images.

Our correction methodology is applied independently to each slice of the multi‐slice dataset. It can be considered as a 2D correction, but a multi‐slice (3D) susceptibility distribution is estimated for each slice. The susceptibility change is estimated once and applied as a temporally constant term while the boiling artifact persists.

**FIGURE 1:**
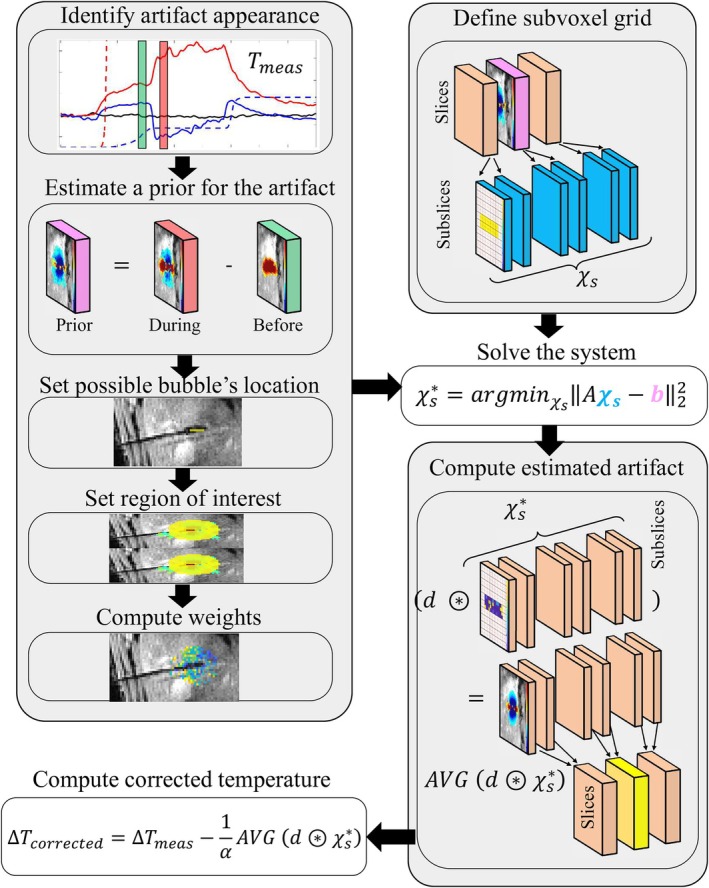
Schematic overview of the correction algorithm. Experimental data are averaged over five dynamic acquisitions before and after bubble appearance and then subtracted from *each* other to estimate a prior of the corrupted temperature distribution related to the artifact appearance. Masks indicating potential locations for the bubbles and the region of interest are then semi‐automatically defined. Weights are computed using magnitude and temperature data, and a subvoxel grid is defined according to the spatial dimensions of the EPI slices. The key contribution is that multiple individual bubbles are allowed in multiple slices of the subvoxel grid, identifying both the location and susceptibility values that better simulate the a priori information. Next, the artifact is finally computed from the estimated susceptibility spatial distribution, followed by regridding it back to the voxel resolution. To obtain the corrected thermometry, this simulated artifact is subtracted from the original temperature images. The symbol for susceptibility in this figure refers to the susceptibility variation introduced by boiling‐induced bubbles, discretized at the subvoxel level.

Figure 1 shows a schematic overview of the correction algorithm. The susceptibility distribution is obtained by solving an inverse problem. The details are described next.

### 3.2 Correction Algorithm—Prior Image

The first step is to identify the representative stable state of the boiling‐induced artifact. We define the following states:

- “Before” state image: Average of five dynamics before the appearance of the boiling‐induced artifact.
- “During” state image: Average of five corrupted dynamics after artifact establishment/stabilization.
- “Prior” estimate image: Difference between the “during” state and the “before” state.

The prior image provides an estimate of the representative stable component of the artifact through time. This is a temperature variation image dominated mainly by the effects of the boiling‐induced artifact. We convert it to RDF, as explained in Section 2.

### 3.3 Correction Algorithm—Masks, Weights, and Data Vector

The following masks and data vector are used in the algorithm.

- Bubble mask $M_{b}$: Identifies possible positions of nonzero susceptibility. This mask is positioned inside the signal‐void of the ablation probe at the beginning of the ablation.
- Region of Interest (ROI) mask $M_{\mathrm{ROI}}$: Covers the ablation zone. To create the mask, a two‐voxel thickness line is marked in the slices to approximately identify the probe position and then dilated by nine voxels. Voxels marked by $M_{b}$ are excluded from $M_{\mathrm{ROI}}$.
- Weight matrix W: Stores for each voxel the inverse of the standard deviation of the RDF. For each voxel marked by $M_{\mathrm{ROI}}$, we first compute a local signal‐to‐noise ratio (SNR) as follows: the magnitude image is averaged over the “during” state, and this average is divided by the standard deviation (in time) of the magnitude values measured before heating is applied. Then, this SNR provides an estimate of the inverse of the standard deviation of the phase [35], which is converted to the inverse of the standard deviation of the RDF (see Supporting Information for details). Otherwise, if not marked by $M_{\mathrm{ROI}}$, the weight is zero.
- Data vector b: generated by applying W and $M_{\mathrm{ROI}}$ to the prior image

### 3.4 Correction Algorithm—Susceptibility and Subvoxel Grid

The subvoxel grid is formed by dividing by 2 the original voxel grid (which includes the slice gaps in the slice‐thickness). For ease of notation, we use the symbol $\chi_{s}$ to describe the susceptibility variation introduced by boiling‐induced bubbles, discretized at the subvoxel level. Nonzero susceptibility is assigned only to subvoxels contained inside voxels marked by $M_{b}$. The RDF induced by the susceptibility variation in the subvoxel grid is simulated by $d⊗\chi_{s}$ (where d is the dipole kernel, see Section 2). The RDF caused by $\chi_{s}$ is obtained at the original voxel resolution by averaging the forward simulated RDF values of the corresponding subvoxels.

### 3.5 Correction Algorithm—Inverse Problem and Corrected Dataset

Let N and $N_{s}$ be the numbers of all voxels and subvoxels in the multi‐slice image, respectively. Mathematically, the matrices $M_{b}$, $M_{\mathrm{ROI}}$, and W are N×N; b is N×1; and $\chi_{s}$ is $N_{s}\times 1$. The matrix A ($N\times N_{s}$) in Figure 1 is given by $A=M_{\mathrm{ROI}}\mathrm{WP}D_{A}D_{s}$, meaning: (i) convolution $D_{s}$ ($N_{s}\times N_{s}$) with dipole kernel d in the subvoxel grid; (ii) voxel‐average, via convolution $D_{A}$ ($N_{s}\times N_{s}$) and projection P ($N\times N_{s}$) to the voxel grid; (iii) weighting W (N×N); and (iv) cropping $M_{\mathrm{ROI}}$ (N×N).

The output $A\chi_{s}$ is compared to the data vector b with a two‐norm least‐squares formulation. The solution provides a subvoxel susceptibility distribution that reproduces in the voxel grid the representative state of the artifact identified by the prior image. Additionally, $\chi_{s}$ values are limited to [0, 10] ppm [24, 28]. The use of least‐squares and disjoint masks is inspired by Liu et al. [36].

The computational code was implemented in Python using NumPy and SciPy libraries. The optimal $\chi_{s}^{*}$ was computed using SciPy's interior‐point solver [37]. The computational time for one slice, using eight cores of one computer with Intel Core i7‐14700K‐CPU@5.6GHz processor and 64‐GB RAM, was 1.3 ± 0.2 s.

Finally, the forward simulated RDF calculated with $\chi_{s}^{*}$ is converted to temperature variation and subtracted from the original measurements during the period when the artifact is detected, obtaining the corrected temperature variations.

### 3.6 Animal Preparation

In vivo experiments were performed in seven pig livers, following European rules for animal experimentation, approved by the ethics committee Comité d'Ethique en Expérimentation Animale of Bordeaux No. 50 (CEEA50, France), and complying with the ARRIVES guidelines. After sedation by intramuscular injection of Ketamine (10–20 mg/kg), acepromazine (0.1 mg/kg), and Buprénorphine (9 μg/kg), the animal was anesthetized with an intravenous injection of Propofol (1–2 mg/kg). It was intubated and ventilated at 15 breaths per minute using an MR‐compatible ventilator (Aestivia, General Electric, Fairfield, CT, USA) and placed in the MRI scanner in the supine position. Continuous breathing of isoflurane (1.5%–3%) in a mixture of air/oxygen 50/50 helped maintain anesthesia. Intra‐arterial pressure and cardiac rhythm were monitored (Carescape, General Electric, Fairfield, CT, USA).

### 3.7 MWA Procedure

Ablations were performed using an AveCure microwave system (MedWave, San Diego, USA). A 14‐gauge antenna was inserted percutaneously under MRI guidance, connected to an external generator via a shielded cable provided by the manufacturer. A 25‐dynamics delay (∼100 s) was observed before starting the ablation. The ablation duration was 7.5 min with a target temperature of 80*°*C. Three to four ablations were performed per animal in different liver locations to avoid lesion overlap.

### 3.8 Acquisition and Image Reconstruction

PRFS‐based MR thermometry was acquired dynamically on 1.5T and 3T MRI scanners (Magnetom Avanto Fit, Aera and Prisma, Siemens Healthineers, Erlangen, Germany) using a multi‐slice multi‐shot GRE EPI sequence, with seven segments, FOV 300 × 300 mm^2^, matrix size 128 × 128, GRAPPA 2, in‐plane spacing 2.3 × 2.3 mm^2^, slice thickness 3 mm, slice gap 1.5 mm; and, for 1.5T/3T respectively, TE 15/11 ms, FA 40/30°, bandwidth‐per‐pixel 815/953 Hz. To freeze the motion, acquisitions were performed under respiratory gating during the most stable part of exhalation (∼1 s), thereby reducing spatial coverage to seven slices. The image update rate is equal to the respiratory period (∼0.2 Hz). The stack of slices was acquired in paracoronal or parasagittal orientation (i.e., rotational invariant to B0‐axis) to minimize through‐plane motion and to locate the microwave antenna in the central slice of the stack. Phase‐encoded direction was right–left or anterior–posterior, respectively. The spine coil integrated into the MRI bed and a loop coil positioned on the abdomen and surrounding the device's insertion point were used for data acquisition. At the end of the experiment, gadoteric acid (0.5 mmol/kg, Clariscan Gé, GE Healthcare) was injected intravenously, and 3D T1‐weighted images were acquired 5 min after injection to visualize the non‐perfused volumes, using: FA 10°, resolution 1.2 × 1.2 × 1.2 mm^3^, FOV 380 × 310 × 125 mm^3^; and, for 1.5T/3T, respectively, TE 2.16/2.2 ms, TR 4.49/4.56 ms. The hyperintense area in T1‐weighted images was manually segmented, and its volume was recorded (“T1w ablation zone”).

### 3.9 Temperature and TD Calculation

Temperature calculation was performed using the PRFS method, followed by a spatiotemporal drift correction [38]. The proposed correction of susceptibility artifacts was then applied.

A temporal first‐order low‐pass Butterworth filter (cut‐off 0.04 Hz) was applied on temperature variations. The initial temperature was set to 37°C. The cumulative TD based on the Sapareto equation [39] was computed, considering an equivalent dose of 240 min at 43°C (CEM43) as lesion (“TD ablation zone”).

### 3.10 Temperature Data and Lesion Size Analysis

Data analysis was performed retrospectively, aimed at assessing the ability of the method to reproduce artifacts and correct the temperature measurement errors. The condition to decide if a bubble‐induced artifact occurred was the presence of negative temperatures variations in two lobes near the probe during ablation, which was checked manually via observation of the multi‐slice temperature distribution through time. Under the considered in vivo conditions, no gold‐standard information was available via temperature probes. Therefore, qualitative comparisons of spatiotemporal temperature distributions are shown.

To demonstrate the feasibility and advantages of this methodology under actual treatment conditions, comparisons were performed with the postprocedural “T1w ablation zone” against two “TD ablation zones”: one given by the uncorrected data and another obtained with the correction methodology proposed in this work. T1w ablation zones were manually segmented using 3DSlicer [40] after a B1‐field inhomogeneity correction with ITK‐N4 [41]. As the two sequences were triggered at different respiratory phases and stages of the experiments (between 15 min and 3 h after ablation), the “TD” and “T1w” ablation zones presented a spatial offset. The TD data were aligned automatically using rigid registration [42] and regridded at T1w image resolution. The volumetric Dice score, Total Overlap (TO), False Negative Rate (FNR) [43, 44], volume of lesion and length of its three main axes were computed with the ANTs library [42]. The statistical significance of the improvement after correction was computed with the Wilcoxon signed‐rank test.

The B0 direction in all temperature maps shown in this work is bottom‐to‐top.

### 3.11 Numerical Simulation

A subvoxel grid was defined with 1.15 × 1.15 × 2.25 mm^3^ resolution and a susceptibility map was set to simulate the effect of a boiling‐induced artifact in the two central and four adjacent subslices. This input artifact was simulated with the subvoxel kernel and averaged to create artificially corrupted temperatures at a voxel resolution of 2.3 × 2.3 × 4.5 mm^3^ (Figure S2). The underlying assumptions were that bubbles can be smaller than the voxel size, and that the effects of the susceptibility located at adjacent slices are relevant.

The central slice artifact was used as input for the inverse problem to be solved by three different methods. Method 1 (voxel grid and central slice 𝜒 distribution): uses voxel grid and nonzero susceptibility restricted to the central slice. Method 2 (subvoxel grid and central subslices 𝜒 distribution): uses subvoxel grid and nonzero susceptibility restricted to the central subslices. Method 3 (subvoxel grid and central and adjacent subslices 𝜒 distribution): uses subvoxel grid and nonzero susceptibility of the central and adjacent subslices.

The objective is to compare the temperature distribution of the estimated artifact versus the simulated artifact. The root mean square error (RMSE) and the absolute difference between the simulated and estimated temperature artifacts were computed for a single time over a ROI of 10 × 10 voxels covering the artifacts, after exclusion of the two centered lines (where the center susceptibility voxels are located). The three different methods were compared only on this numerical simulation. Method 3 was then used on all experimental data.

## 4 Results

**FIGURE 2:**
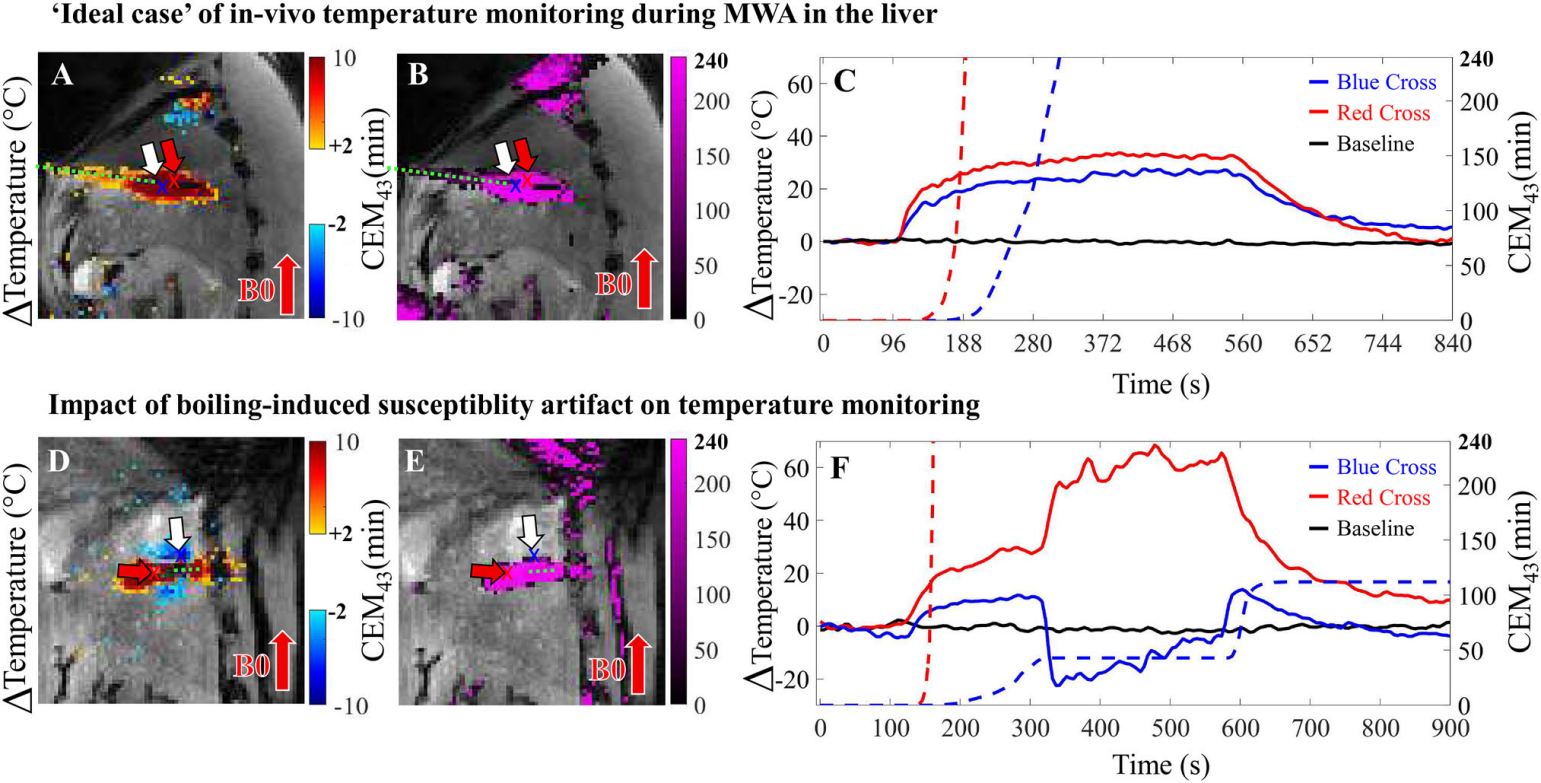
Representative case of liver MWA in an in vivo porcine model with and without the presence of boiling‐induced susceptibility artifacts. In both cases, the B0 field is oriented in the bottom‐top direction of the images. The left panel shows temperature (A, D) and thermal dose (B, E) maps overlaid on magnitude images, along with an approximate marking of the ablation probe position (dotted green line). Temperature maps represent temperature variation and have a cut‐off for low variation (−2°C to 2°C). The right panel shows the corresponding temporal evolution in two voxels indicated by blue and red arrows for the temperature (full line) and thermal dose (dashed line) for a case at 3T (C, top) and 1.5T (F, bottom). The top line (pig 7, abl. 1) shows a physically realistic spatial and temporal temperature distribution. The bottom line (pig 1, abl. 3) showed unphysical negative temperature values. The blue line in (F), corresponding to a voxel in the negative lobe of the dipole artifact, suddenly drops in temperature, and the voxel never reaches the theoretical CEM43 240 min threshold of cell necrosis (dashed blue line), although its location is very close to the ablation probe. On the contrary, the voxel in the positive lobe of the dipole artifact registers a sudden “temperature jump” after the appearance of the artifact and overestimates the maximum relative temperature of 30°C (solid red line in F).

State of the art ablation in a physically realistic situation assumes temperature increases during ablation and returns to its baseline after the procedure (Figure 2, first row). In the case of bubble formation (Figure 2, second row), susceptibility artifacts have a “butterfly” shape and induce unrealistic temperature variations in the range of ±30°C/40°C. We report very heterogeneous sizes and shapes obtained with 1.5T and 3T (Figure S3).

**FIGURE 3:**
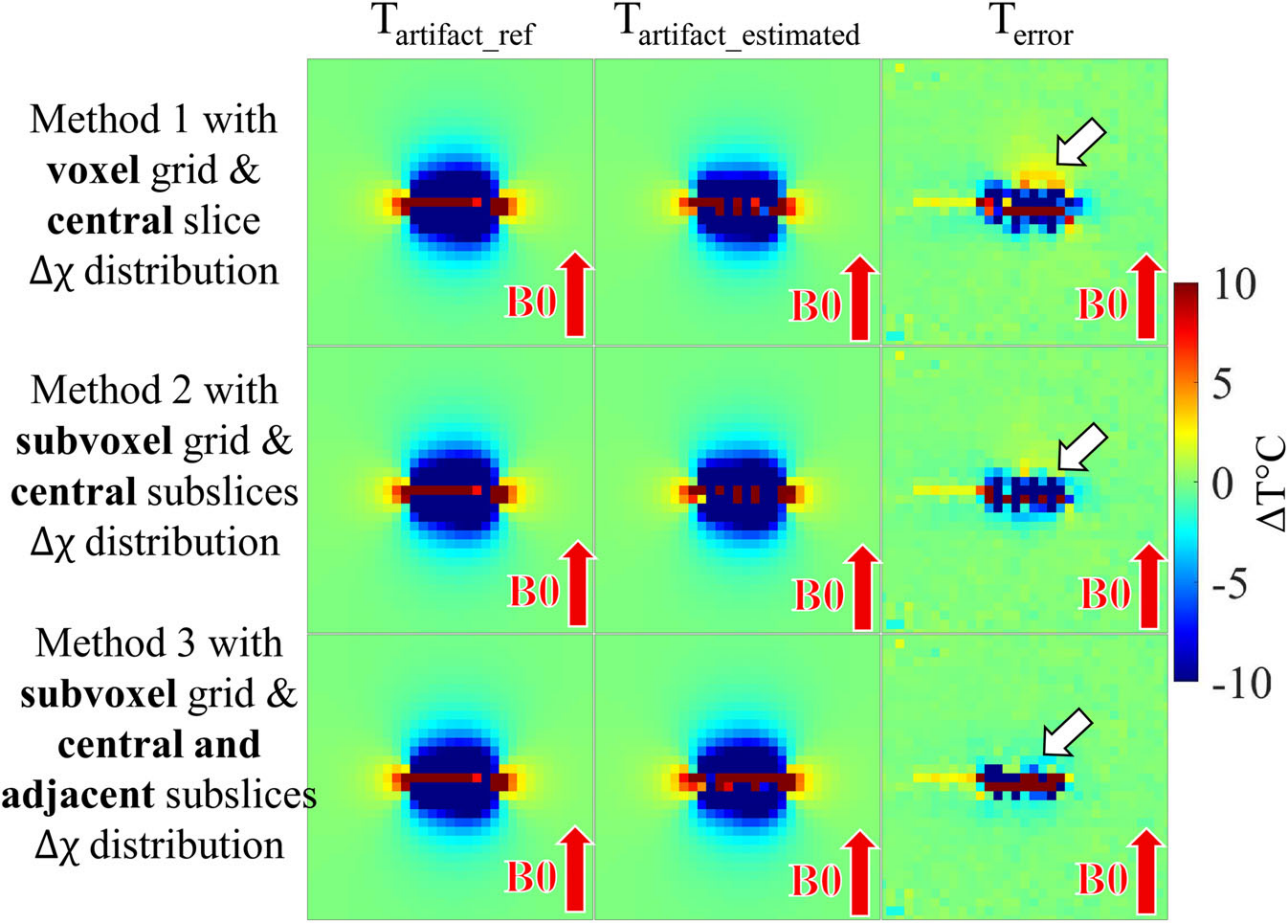
Numerical simulation: result of the optimization problem for the three different methods. See Section 3 for details. Only the central slice is shown, and each row corresponds to a method. The first column is the input simulated artifact; it is the same for the three methods. The second column is the estimated artifact after the susceptibility identification, expected to match the simulated artifact in the first column. The third column is the error between them. Notice how Method 1 underestimates the artifact and leads to temperature errors up to 10°C (white arrow). Method 2 and 3 are progressively better, with significantly less residual in Solution 3 and no sharp temperature spikes in the indicated region.

The three methods used in the numerical simulations resulted on different temperature artifacts (Figure 3), generated by the corresponding estimated susceptibility distributions (Figure S4). Method 1 has temperature errors around −16°C and temperature spikes up to 10°C. Methods 2 and 3 present similar agreement at a distance from the centerline, but Method 3 has smaller temperature errors near the centerline, especially considering the scale of ±2°C (green color), and it does not exhibit temperature spikes. The mean ± SD of the absolute error for Methods 1, 2, and 3 were, respectively, 9.0°C ± 37.0°C, 7.6°C ± 9.2°C, and 3.2°C ± 13.3°C while the RMSE was 2.4°C, 1.9°C, and 0.9°C. Now, for the following results with experimental data, only Method 3 was applied.

Out of seven pigs with 23 MWA (Table 1), 3 (13%) MR temperature acquisitions were excluded due to low SNR, presence of folding artifacts, radiofrequency artifacts or all three. Susceptibility artifacts caused by bubble formation were observed in 11 (48%) acquisitions. The average ± SD time interval between ablation and administration of the contrast agent was 88 ± 49 min with a minimum/maximum of 14/199 min.

**TABLE 1:** Summary of in vivo preclinical experiments.

Swine	Ablation	Artifact?	Dice uncorrected (%)	Dice corrected (%)
Pig 1 (1.5 T)	Abl. 1	No	57	—
	Abl. 2	Yes	72	75
	Abl. 3	Yes	75	75
	Abl. 4	Yes	59	61
Pig 2 (1.5 T)	Abl. 1	NA	NA	NA
	Abl. 2	No	43	—
	Abl. 3	Yes	61	60
Pig 3 (1.5 T)	Abl. 1	Yes	61	62
	Abl. 2	No	42	—
	Abl. 3	Yes	48	50
	Abl. 4	No	64	—
Pig 4 (1.5 T)	Abl. 1	Yes	62	65
	Abl. 2	Yes	63	62
	Abl. 3	No	61	—
Pig 5 (3.0 T)	Abl. 1	No	58	—
	Abl. 2	No	70	—
	Abl. 3	Yes	69	72
Pig 6 (3.0 T)	Abl. 1	Yes	60	66
	Abl. 2	NA	NA	NA
	Abl. 3	NA	NA	NA
Pig 7 (3.0 T)	Abl. 1	No	61	—
	Abl. 2	Yes	65	65
	Abl. 3	No	74	—
Mean			63	65
Median			62	65
IQR			7	8

*Note*: Twenty‐three microwave liver ablations were performed over seven pigs. The swine and its ablations are numbered in the first and second columns (referenced throughout the text), along with the respective B0 magnitude. The presence of boiling artifacts is marked on the third column (11 cases with “yes”). Three cases were excluded (not applicable [NA]) due to low signal‐to‐noise ratio limiting interpretation of thermometry and thermal dose volume. Volumetric Dice coefficients are displayed for comparisons between thermometry data and T1w postprocedural data: fourth column, Dice for uncorrected‐and‐T1w; fifth column, Dice for corrected‐and‐T1w. Mean, median, and interquartile range (IQR) were computed for the Dice of the 11 cases that had artifacts.

**FIGURE 4:**
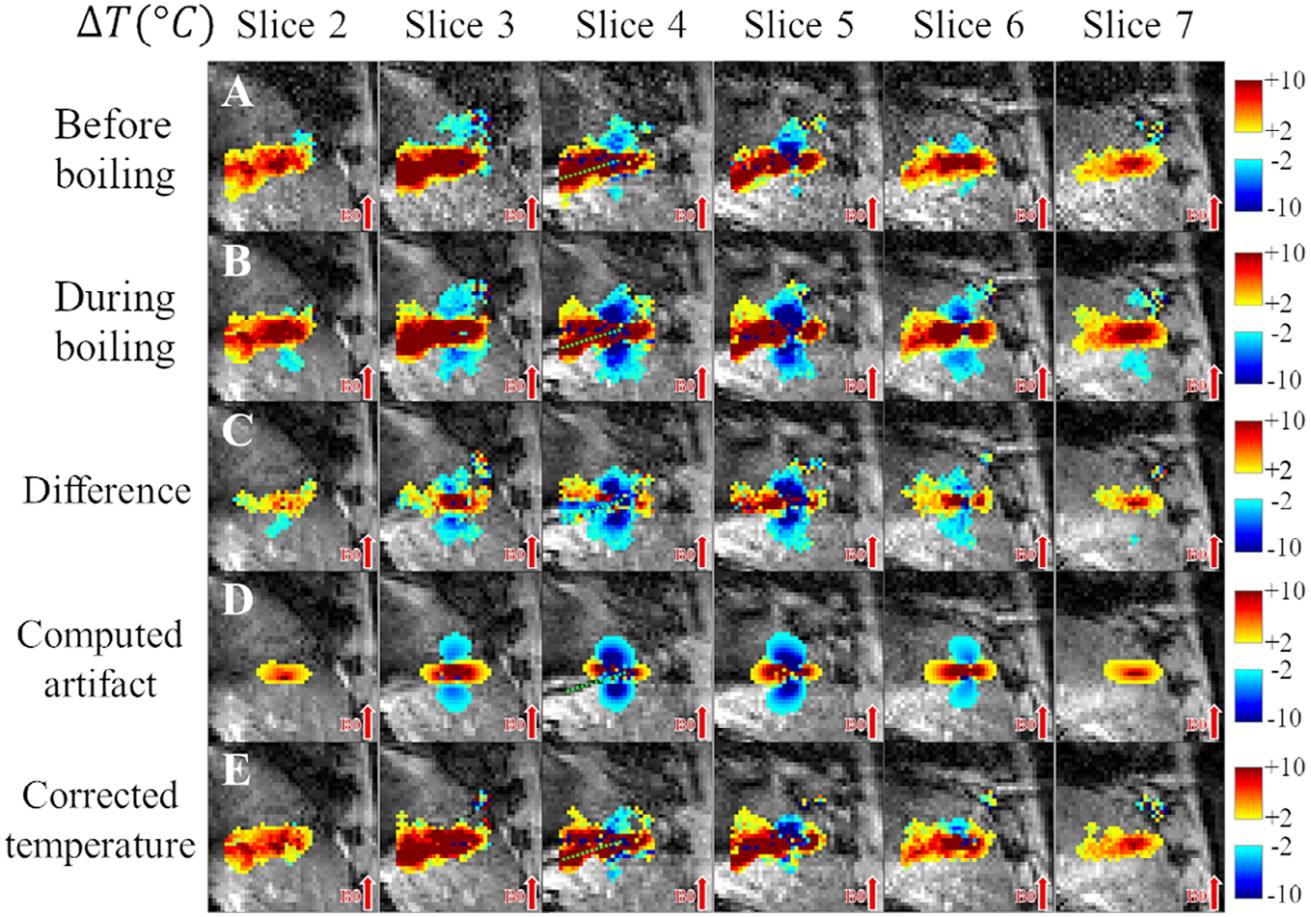
Spatial distribution of temperature in one of the in vivo liver ablation experiments (pig 6, abl. 1) before and after correction. Temperature maps of six adjacent slices are overlaid on magnitude images with a cut‐off between −2°C and 2°C. An approximate marking of the ablation probe position is given by a dotted green line. Temperature maps before (A) and during (B) boiling‐induced susceptibility artifacts. (C) A priori image of corrupted temperature distribution related to the artifact appearance. (D) Estimated artifact. (E) Corrected temperature maps. Notice the appearance of the dipole‐like artifact on row C, which shows the states “during boiling” with negative temperature values lower than −10°C. Row E presents the corrected temperature maps for this experiment, after the simulated artifact has been subtracted; notice the similarity with row A (non‐corrupted data). Comparison of Rows C and D shows how the computed artifact tries to replicate detailed features found in the artifact in data (e.g., distorted dipole shapes with angular features). The negative temperatures visible in Row A (“before boiling”) indicate that the images are already affected by a susceptibility artifact. This artifact was present since early in the ablation, possibly caused by blood flow or gradual gas bubble formation. Nevertheless, it is followed by a very clear and bigger dipole‐shape artifact. This second, most impactful and clear artifact, is the one that is corrected in this example.

Figure 4 presents the correction for pig 6, abl. 1. The prior images register negative temperature variations as low as −44°C (Slices 4 and 5) and positive variations up to 23°C (other slices). Comparison with the computed artifact demonstrates the ability of the proposed methodology to simulate the representative state of the artifact over all slices, even in the vicinity of the MW probe. Figure S5 exemplifies another case (pig 1, abl. 3) of successful correction, where temperature errors induced by the boiling artifact range from −41°C (Slices 1 and 2) to 35°C (Slices 3 and 4).

**FIGURE 5:**
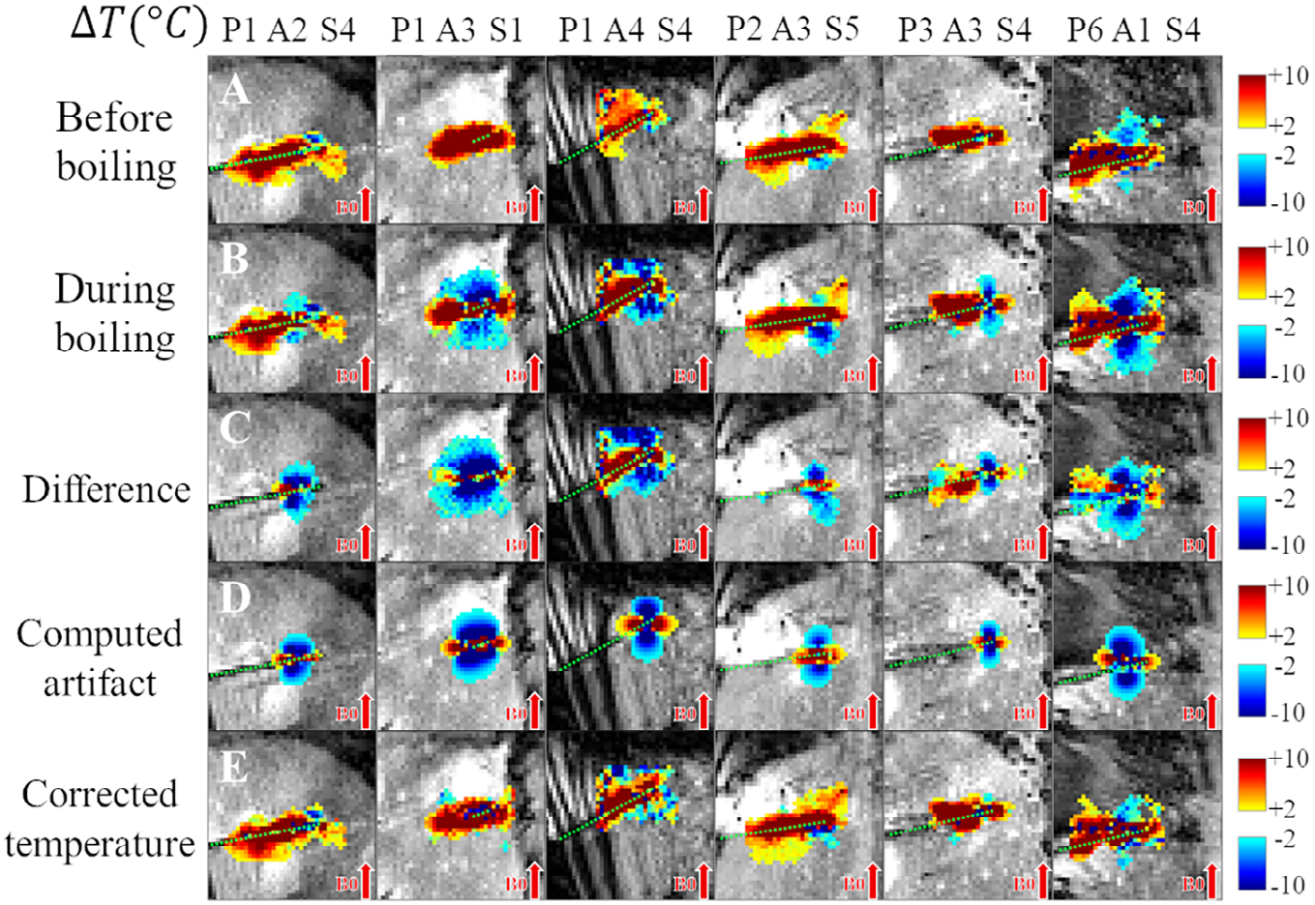
Spatial distribution of temperature in six different in vivo liver ablation experiments before and after correction. Top of figure: P = pig, A = ablation, S = slice. Temperature maps of six experiments are overlaid on magnitude images with a cut‐off between −2°C and 2°C. For each experiment, the depicted slice is located on the MW probe; except for the one in the second column (P1 A3 S1) because in this case the ablation probe went through the slices at an angle (its depicted slice is positioned at the tip of the probe). An approximate marking of the ablation probe position is given by a dotted green line. Temperature maps before (A) and during (B) susceptibility artifacts. (C) A priori of corrupted temperature distribution related to the artifact appearance. (D) Estimated artifact. (E) Corrected temperature maps. The comparison of Rows C and D highlights the similarities between the artifact appearing in the data and the computed artifact. Also compare Rows A and E, marking the quality of the correction even at close vicinity of the MW probe.

Figure 5 presents a selection of corrections over many experiments to display how the proposed methodology works in scenarios with varying degrees of artifact intensity, in‐plane orientations of the dipole‐like structure with respect to the ablation probe, shapes of the artifact, and B0 magnitudes (1.5T and 3T).

**FIGURE 6:**
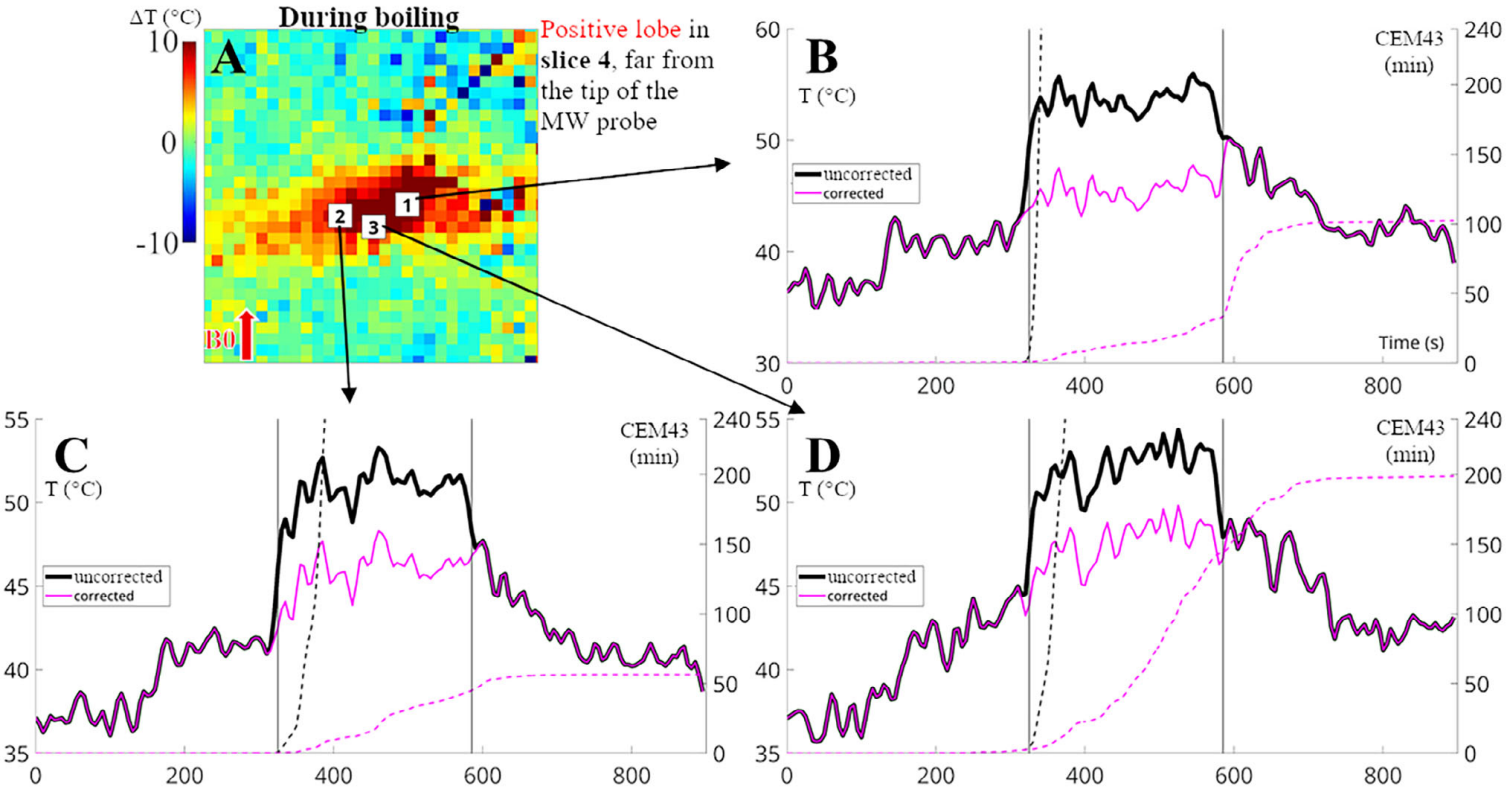
Temporal evolution of the temperature for Slice 4 of in vivo liver ablation experiment pig 1 Abl. 3, before and after correction. The temperature colormap shows temperature variation, while the time plots show the variation plus an offset of 37°C. (A) Temperature map during boiling‐induced susceptibility artifact. (B–D) Temperature evolution (full lines) and CEM43 (dashed lines) are plotted for three voxels marked on (A). Vertical lines indicate when the correction starts and ends. The tip of the ablation probe for this experiment is mostly located on Slice 1 (see Figure S5), and this figure depicts Slice 4, thus the effect of the artifact is seen here mostly as a positive lobe, overestimating the temperature measurements. After correction, the “temperature jumps” are eliminated, and the time evolution returns to the general trend and scale seen before the appearance of the artifact.

Figure 6 compares temperatures and TD time evolutions for Slice 4 of pig 1, abl. 3. In this experiment, Slice 4 is far from the probe's tip (Slice 1), thus the measurements in this region are impacted by the positive lobe of the artifact. The marked voxels show temporally stable temperature “jumps/offsets” around 10°C that are removed after the correction, impacting the dosimetry. The region was classified as non‐ablated after correction, agreeing with postprocedural T1w (Figure S6). Figure S7 also shows time evolution comparisons for this experiment for Slice 1. In Voxels 1, 2 and 3, temperature “jumps” of −30°C, +7°C, and −15°C, respectively, were corrected, which impacted the lesion estimate: Voxels 1 and 3 now reach the TD threshold, and Voxel 2 no longer does.

**FIGURE 7:**
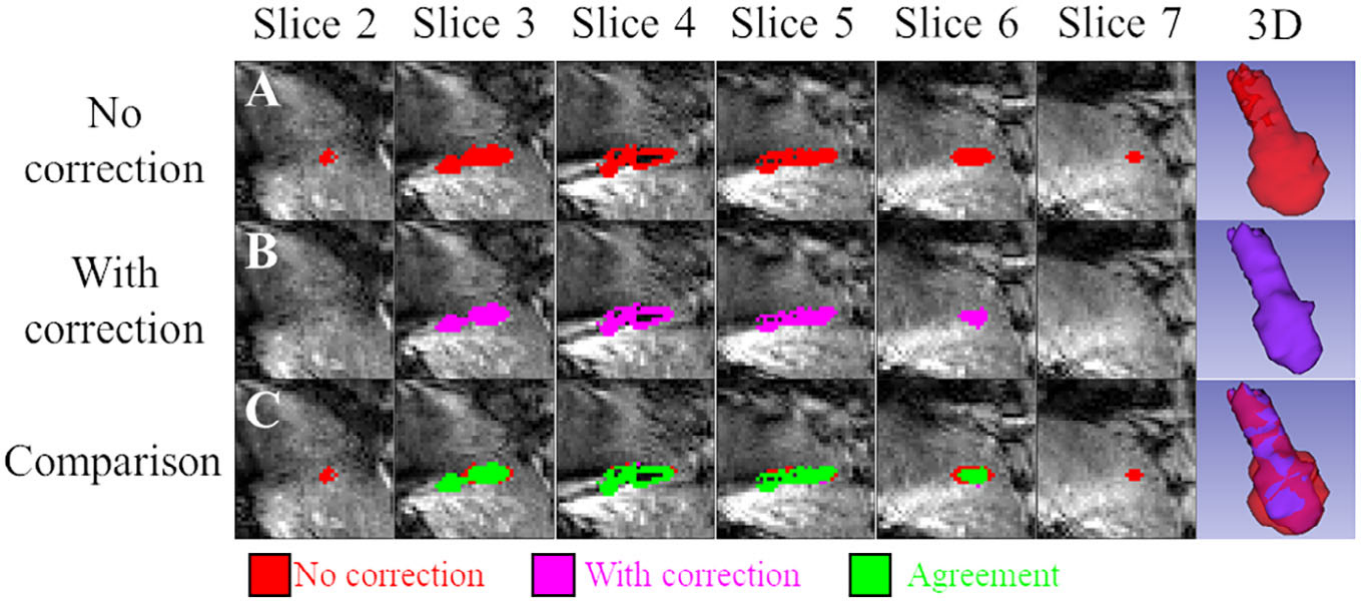
A case study (pig 6, abl. 1) comparing the impact of the correction on dosimetry. In seven consecutive slices, the estimated lesion size from CEM43 computation is overlaid over the magnitude images. A 3D representation of the lesion volume is displayed in the last column. The initial row represents the uncorrected data set (red), the second row depicts the proposed correction (purple), and the final row illustrates the degree of agreement between the two methods (green).

The impact of multi‐slice TD estimation is exemplified in Figure 7 (pig 6, abl. 1). The correction estimated no lethal damage in Slices 2 and 7, indicating a 9‐mm reduction (two 4.5‐mm thickness slices) of the predicted lesion size along the direction perpendicular to the slice plane. The spatial extent of the lesion in Slice 6 was also reduced by two voxels in the top‐to‐bottom and left‐to‐right directions (∼4.6 mm each). The slices closer to the probe (3–5, and) only experienced minor changes: one or two voxels (∼2.3 and ∼4.6 mm). Thus, overestimation is more prominent in the slices farther from the MW probe. In fact, temperatures near the probe were very high (∼80°C), requiring a short time to be considered destroyed (0.11 s in 60°C for 240 CEM43). Therefore, deviations due to the artifact in voxels close to the ablation probe can be very apparent in temperature monitoring, but not necessarily on TD estimation. Far from the probe, the artifact has a more significant impact on TD because the original temperatures were below 60°C. Figure S8 (pig 1, abl. 2) and Figure S9 (pig 1, abl. 3) present further TD comparisons, where similar reductions of the predicted lesion size were found.

**FIGURE 8:**
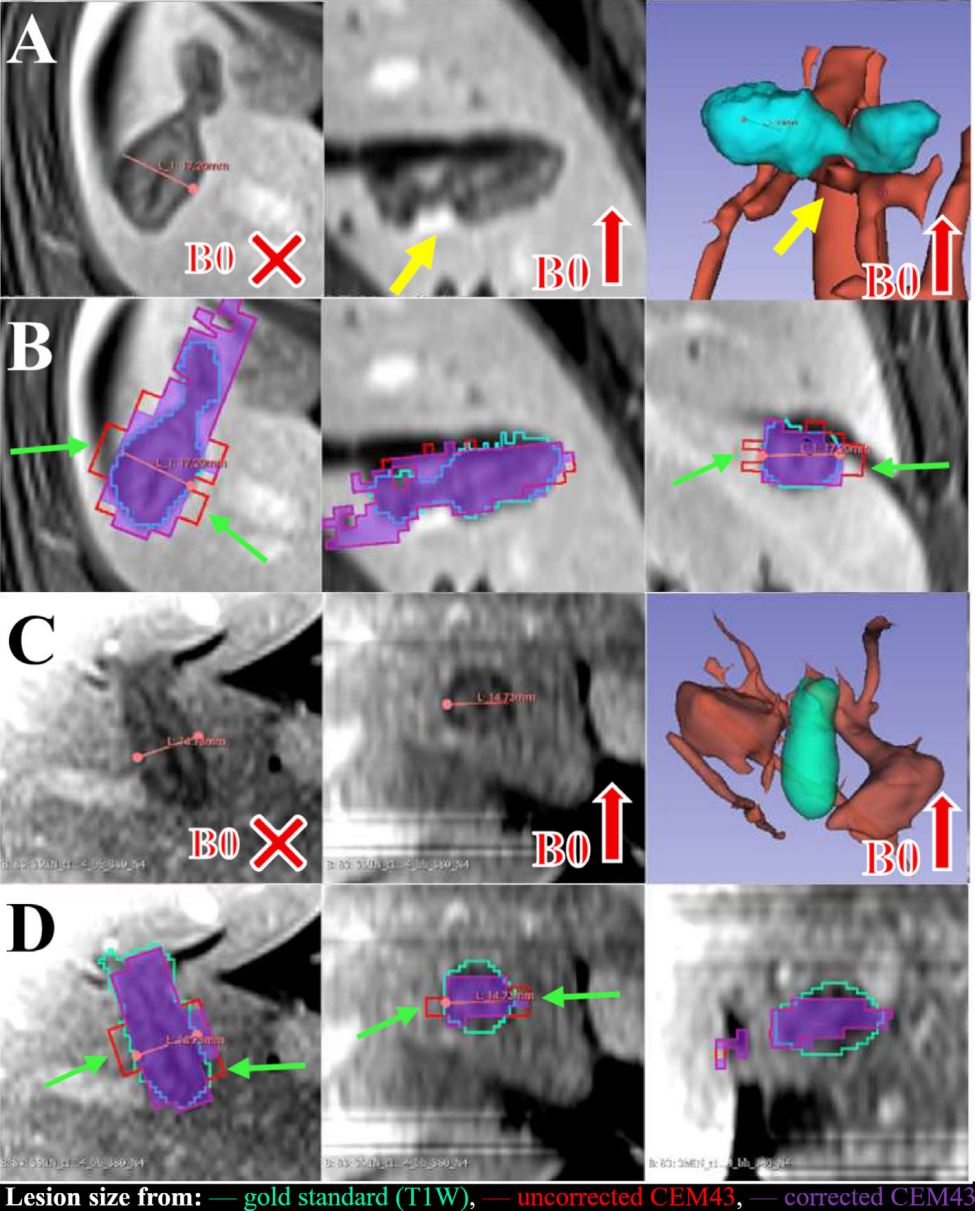
Impact of the correction on dosimetry: A comparison of TD versus T1w ablation size. The reference T1w ablation zones (cyan) and relevant vascularization (orange) were segmented using 3DSlicer after a B1 inhomogeneity field correction. Post‐contrast T1w images and T1w ablation zones are displayed for two representative case studies: (A) pig 6, abl. 1; (C) pig 1, abl. 2. For each experiment, 3D lesion size comparisons from TD before (red) and after (purple) correction are overlaid on post‐contrast T1w images (B, D). An overestimation of lesion size of 4.5 mm to 9 mm (green arrows) in the minor axis perpendicular to the main axis of the lesion is visible (green arrows). Additionally, a heat sink effect is well illustrated (yellow arrow) in (A, B) with reduced ablation size using either TD or T1w estimation at the vicinity of the vessel. Such an effect is not observed in (C, D) because the vessels were located further away from the MW probe in this case.

Figure 8 presents T1w ablation zones versus TD ablation zones with and without correction. The thermally damaged region appears in hypointense with sharp edges. The first case shows a distorted lesion shape due to an adjacent blood vessel, while the second case shows an ellipsoid‐shaped lesion. Without correction, overestimation of the lesion size up to 9 mm (two 4.5‐mm thickness slices) in the minor axis was observed. Corrected maps show better agreement with the T1w ablation zone. For Figure 8A,B (pig 6, abl.1), uncorrected TD volume and Dice were 10.2 cm^3^ and 0.60, respectively, while correction changed it to 8.1 cm^3^ and 0.66. For Figure 8C,D (pig 1, abl. 2), uncorrected values were 6.2 cm^3^ and 0.72, against 5.8 cm^3^ and 0.75 after correction, respectively.

**FIGURE 9:**
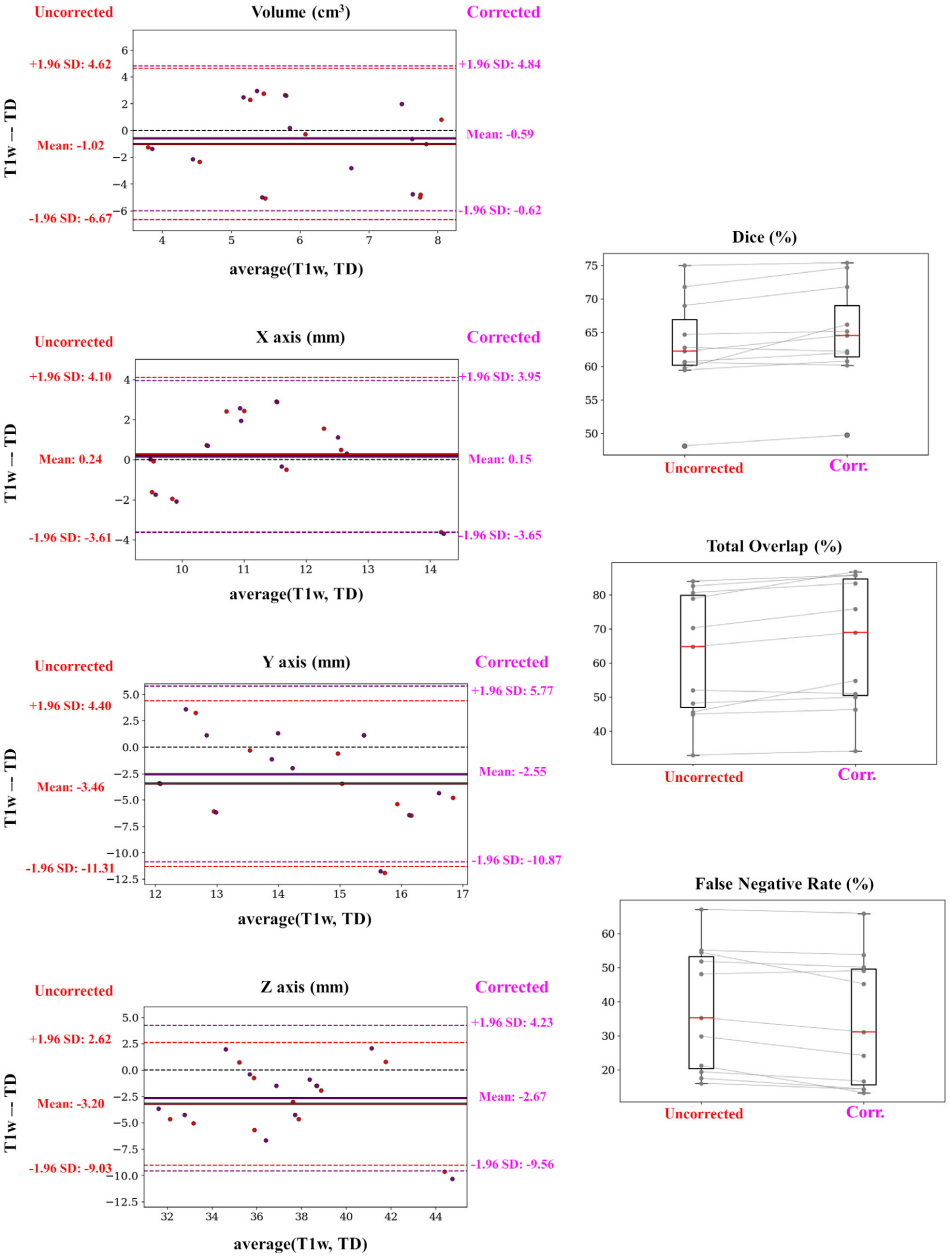
Statistics of volumetric geometrical and overlap measures. Bland–Altman plots in the first column for the lesion volume and its three main axes. Boxplots in the second column for the Dice score, Total Overlap and False Negative Rate. The comparison was computed between the lesion in the T1w postprocedural images with two thermal dosage lesions: one based on uncorrected ablation data (red) and the other with corrected data (purple).

The volumetric effect of the correction over TD for all 11 cases is presented in Figure 9. The comparison is performed using T1w postprocedural information against uncorrected and corrected TD. The overlap metrics indicate higher agreement between corrected‐and‐T1w measures compared to uncorrected‐and‐T1w measures: median volumetric Dice increased by 4.8% (0.62–0.65, *p* = 0.005), along with 6.2% increase for median TO (0.65–0.69, *p* < 0.001), and 11.4% reduction for median FNR (0.35–0.31, *p* < 0.001). The median geometric metrics also indicate improvement on the agreement, although the changes were not statistically significant: median overestimation of lesion volume (T1w‐TD) reduced from −1.03 to −0.62 cm^3^ (40% reduction, *p* = 0.259), most noticeable in *Y*‐axis (42% reduction in the median from −3.44 to −1.98 mm, *p* = 0.416) and *Z*‐axis (50.5% reduction in the median from −3.03 to −1.50 mm, *p* = 0.483). All metrics are summarized in Tables S1 and S2.

## 5 Discussion

This study—using a commercially available multi‐slice multi‐shot EPI sequence—investigated the impact of a new correction methodology for boiling‐induced susceptibility artifacts, aiming at mitigating bias in MR‐temperature estimation during MWA.

Multi‐slice acquisition is critical for improving visualization of the boiling‐induced artifact and understanding its impact in temperature monitoring. Three factors are relevant:

1. *B0 orientation with respect to the image axes*. For the dipole‐shaped artifact caused by bubbles, the negative temperature lobes align with the B0 direction [24, 45]. This is consistent with the dipole kernel formula and our experimental data.
2. *Orientation of the ablation probe with respect to B0*. This factor impacts visualization, because it changes how the temperature distribution is oriented with respect to the artifact (negative lobes along the probe, or perpendicular, etc.; Figure S10).
3. *Positioning of the slices*. Impacts visualization of the artifact (Figure S11).

The numerical simulations demonstrate an improvement in the correction of a complex artifact when using subvoxels (Method 2) and adjacent slices (Method 3), compared to relying solely on a voxel grid with susceptibility restricted to a single slice (Method 1). Since MRI thermometry is often performed in 2D, a crucial criterion was the ability to estimate artifact effects on adjacent slices using a dedicated multi‐slice methodology. Errors of less than ±2°C are required for correct TD estimation [46], which was the chosen accuracy. This is a reasonable assumption considering the ±30°C–40°C errors due to boiling artifacts. Although the three methods offer reasonable estimates with RMSE of less than 2°C, Method 3 (subvoxel grid and nonzero susceptibility restricted to the central and adjacent subslices) was the most reliable in the numerical simulation, especially when near the centerline.

Our methodology recovers the quality of the measurements evidenced “before” boiling up to an error of ±2°C. The multi‐slice aspect required for the correction is emphasized. For Figure 4 (pig 6, abl. 1), the ablation probe was near Slices 4 and 5, and the multi‐slice acquisition allowed visualization of positive lobes of the artifact in slices farther from the probe (2 and 7), which originally overestimated the TD, and that are corrected by our methodology.

The methodology was able to reproduce the intricate details in the deformed dipole artifact shapes observed in data: inclined/angulated components in the negative lobes (Figures 4 and 5), even though the B0 direction was bottom‐to‐top. This flexibility in artifact reproduction was possible for a diverse set of successfully corrected in vivo experiments (Figure 5).

The validation of lesion size prediction was performed in 3D, which is rare in MR thermometry [47]. We observed improved correspondence between corrected TD and T1w lesions, as evidenced by closer geometric measures, higher Dice and TO, and lower FNR. The geometric measures were more strongly affected in the directions perpendicular to B0 than in the direction parallel to B0, which can be impactful for the scale of change of the scores since all of them were computed volumetrically. The improvement was statistically significant for the median Dice, TO and FNR, but not for the median geometric measures, since no significant improvement was noticed in the overall variability (standard deviation) of the geometric measures among the data points. Nevertheless, overall 3D Dice score (0.65) should be improved for clinical use, but to our knowledge there are no equivalent measurements in the literature under similar conditions (multi‐slice, in vivo, liver, MWA). There are several reasons for this insufficient Dice. First, the *k*‐space sampling of the commercial multi‐shot EPI sequence is not optimized for moving organs, as it is acquiring *k*‐space lines from different slices during one shot, instead of acquiring one slice after another. This leads to within‐slice data from different time points, low SNR and folding artifacts. Second, the major difference between native spatial resolution of real‐time TD and postprocedural T1w images leads to significant partial‐volume effects, requiring dedicated interpolation methods [47]. Lastly, a noticeable underestimation of the lesion in one of the main axes (parallel to B0) is visible even after correction (Figure 8); hence further improvement is needed in recovering the temperature on the negative lobes of the dipole far from the needle.

Our methodology differs from previous works in this subject in two main ways. First, the methodology corrects multi‐slice data, instead of selected slices, allowing for volumetric/3D observations. Second, no specific shape of susceptibility is required to be selected a priori (formulations are usually infinite cylindrical [28] or single sphere [27]), allowing for a distribution in the region of possibilities (e.g., Figure S4). It becomes more easy to handle the susceptibility “source region.” In the usual formulations, a single value of susceptibility is estimated for a selected region, thus, the choice of size/position for this region is very impactful. In our approach, $M_{b}$ can be freely overestimated in size, because many of the voxels marked in $M_{b}$ will still end up with negligible susceptibility. The downside of a bigger mask is the increased computational time. The main advantage is being able to reproduce a wider range of artifact shapes.

The use of subvoxels was also motivated to mitigate a pitfall in the dipole model: for positions near the points of nonzero susceptibility, the kernel d induces deviations that grow without bounds. This is known in the quantitative susceptibility mapping (QSM) literature [33], because its derivation [30] is equivalent to using the first term [33] of a multipole expansion, expected to work only for regions far from the susceptibility source. In the context of boiling‐induced artifacts, sharp variations of susceptibility in space (individual bubbles) will augment this difficulty due to discretization errors. Therefore, a finer discretization is required to comply with the fundamental assumptions of the problem, and it also allows for solutions where the nonzero susceptibility subvoxels are not directly adjacent to the data voxels, alleviating the boundary limitations known for background field removal [30, 36].

The subvoxels do make the inverse problem more difficult since the problem becomes more ill‐posed: multiple distributions can be found that will generate the same artifact (i.e., non‐uniqueness and stability of the solution might not be guaranteed). Future improvements must focus on regularization techniques that use physical information concerning the susceptibility spatial configuration: for example, recent work [48] suggests that signal loss in MR cavitation imaging is caused by susceptibility changes that depend on the diameter of microbubbles; such an imaging method could help to better characterize these artifacts.

Our proposed correction method was applied per‐slice of data (one 3D susceptibility distribution was estimated for each 2D slice of data). Figure S12 shows a correction obtained for case pig 1, abl. 2 using an alternative implementation (“all‐slices”) where a single 3D susceptibility distribution was estimated to correct all slices of data simultaneously (only one inverse problem was solved). Our method can also be used in this “all‐slices” manner, in which case the problem is less ill‐posed because more information is available. However, it is also shown in Figure S12 how the per‐slice correction achieved a better result than the “all‐slices” one, replicating the shape of the boiling‐induced artifact more reliably. A reliable “all‐slices” correction would require addressing additional challenges that were beyond the scope of this work and which are alleviated by the per‐slice implementation: (i) instabilities restricted to one slice (e.g., blood‐flow artifacts; higher noise level) may corrupt the susceptibility estimation that would otherwise work for the other slices if they were treated separately; (ii) the presence of the slice gaps becomes more important. These issues require dedicated future investigations.

The prior selection regarding the onset of the susceptibility artifact is a potential limitation. A perfect correction would require a time dependent simulation [22], since further significant changes in bubble formation after the prior will lead to an inaccurate estimate. However, two conditions mitigate potential errors here. First, the artifact had a stable component (Figure S1), with only small changes through time. Our approach aimed at correcting this stable state, which was the dominant part of the temperature errors introduced in the measurements. Second, the ablation device had a feedback loop to stabilize heating at a target temperature. Bubble formation occurred rapidly and mostly when the setpoint temperature had been reached, therefore, temperature variations were limited when bubble formation occurred and right after its formation.

In terms of automated clinical use, the manual detection of the boiling‐induced artifact is a limitation. In the Supporting Information, we propose a preliminary detection algorithm based on counting voxels in the negative temperature lobes of the artifact along the B0 direction, which achieved 80% success in detection for this dataset (see Figures S13 and S14, and text in the Supporting Information for details).

Our implementation required around 1.3 s to correct each slice. Each slice can be corrected independently in our methodology (i.e., simultaneously in parallel computations). This is less than the repetition time of around 3–4 s (linked to the respiratory phase during anesthesia), therefore, it is suited for real‐time use, only requiring measurements right before the appearance of the artifact and after it stabilizes. However, real‐time usage would also require automatic detection of the needle position. Such information could be derived from MRI images either from the thermometry sequence or from previous 3D or interactive sequences.

In this dataset, the appearance of the bubble‐induced artifact was very sudden (9 out of 11 ablations), but a few (2) gradual cases were observed: pig 5, abl. 3 (Figure S15); and pig 6, abl. 1, in which a transient artifact may have been caused by blood flow (Figure 8). In all cases, the sudden boiling artifact was corrected, which was noticeable by clear and impactful dipole shapes. Two generalizations were out of the scope of this paper, requiring further investigation: (i) the gradual bubble disappearance during cooling (Figure S7); (ii) the presence of transient artifacts caused by motion, blood flow or gradual bubble formation.

## 6 Conclusions

Boiling‐induced susceptibility artifacts significantly corrupt temperature monitoring of interventional ablation via MRI, turning TD prediction locally unreliable. A methodology was designed to correct multi‐slice temperature data, based on the generation of a susceptibility distribution over a finer resolution. The methodology was evaluated in preclinical in vivo experiments, recovering temperature images, and enabling better lesion estimation—validated with T1w postprocedural images.

## Conflicts of Interest

Valéry Ozenne and Bruno Quesson are co‐founders/shareholders of Certis Therapeutics. Pierre Bour and Thibaut Faller are employees of Certis Therapeutics.

## Supporting information


**Data S1:** mrm70129‐sup‐0001‐supinfo.pdf.
