# Supplementary material for: A Subvoxel Correction of Boiling‐Induced Susceptibility Artifacts in Magnetic Resonance Thermometry and Dosimetry for Monitoring Microwave Thermoablation: A Feasibility Study in a Swine Model
Source: Magn Reson Med. 2025 Oct 16;95(3):1698–713. doi: 10.1002/mrm.70129 (PMC12746378; doi:10.1002/mrm.70129)
Supplement: Supplementary file 1 — Data S1: mrm70129‐sup‐0001‐supinfo.pdf. [file MRM-95-1698-s001.pdf]

Supplementary Material

Methods – Weights in the inverse problem

For each voxel in the ROI mask M_{ROI}, a local SNR value is computed as

$$SNR = \frac{Mag}{\sigma_{Mag}},$$

where Mag refers to the value of the magnitude image averaged in the "during" state, and σ_{Mag} is the standard deviation of the magnitude computed for each voxel before heating was applied. Based on the work of Conturo et al[1], the standard deviation of the phase (σ_{ϕ}) is proportional to $\frac{\sigma_{Mag}}{Mag}$. We use this proportionality as an estimate for σ_{ϕ} , therefore:

$$\sigma_{\phi} = \frac{\sigma_{Mag}}{Mag} = \frac{1}{SNR}.$$

This can be aptly converted to the standard deviation of the RDF: $\sigma_{RDF} = \sigma_{\phi}/(\gamma B_0 TE)$. Therefore, the SNR provides an estimate for the inverse of σ_{RDF} :

$$\frac{1}{\sigma_{RDF}} = SNR \cdot \gamma B_0 TE\ .$$

The value $1/\sigma_{RDF}$ is stored in the weight matrix W (see Methods section). Similar versions of this estimate have been employed in the literature[2,3]. For our purposes, the proportionality is sufficient.

The assumptions for such approximation hold even for "fairly small SNR values"[4]. To maintain the assumptions, a number of voxels are set as zero weight in this step (effectively excluded from the problem) if they present: (i) SNR lower than the 5th percentile of SNR in the slice; (ii) magnitude lower than 10% of the 95th percentile of magnitude in the slice before heating.

In terms of the inverse problem, this weight W matrix represents the inverse of standard deviation of the RDF measured at each voxel, where we have the underlying assumptions of normal error distribution (use of least squares) and uncorrelated measurements (W is a diagonal matrix).

References:

1. Conturo TE, Smith GD. Signal-to-noise in phase angle reconstruction: Dynamic range extension using phase reference offsets. *Magnetic Resonance in Med*. 1990;15(3):420-437. doi:10.1002/mrm.1910150308

2. Maier F, Fuentes D, Weinberg JS, Hazle JD, Stafford RJ. Robust phase unwrapping for MR temperature imaging using a magnitude-sorted list, multi-clustering algorithm. Magn Reson Med. 2015 Apr;73(4):1662-8. doi: 10.1002/mrm.25279. Epub 2014 May 8.

3. Rieke V, Butts Pauly K. MR thermometry. J Magn Reson Imaging. 2008 Feb;27(2):376-90. doi: 10.1002/jmri.21265.

4. Gudbjartsson H, Patz S. The Rician distribution of noisy MRI data. Magn Reson Med. 1995 Dec;34(6):910-4. doi: 10.1002/mrm.1910340618. Erratum in: Magn Reson Med 1996 Aug;36(2):332.

Discussion – Preliminary detection algorithm for boiling-induced artifacts

To investigate the feasibility of automating our approach, a novel detection algorithm was designed to detect, at each time step, the occurrence of the boiling-induced artifact. This detection algorithm was tailored for the experimental conditions of this work.

We consider three fundamental assumptions of experimental conditions:

1. The slices are placed in paracoronal or parasagittal orientation (i.e., rotational invariant to B0-axis) so that the B0 direction is in one of the main axes of the image (vertical or horizontal). The ablation needle is parallel to the plane of the slices. The algorithm is applied to the so-called 'central slice' through which the needle passes.

2. The central slice, as well as the tip and entry point of the needle, can be identified using two markers in space.

3. The duration of the ablation was considered known (in seconds).

The following steps were applied using MATLAB and are displayed in Figure S13. We performed step A manually. All the other steps (B, C, and D) are automatic.

A. **Position selection (manual):** The entry point and tip of the ablation needle were manually marked, using the first magnitude image from the

beginning of the EPI sequence (i.e., before the ablation period). The imaging plane of these markers is the central slice.

B. **ROI definition (automatic):** A line was drawn through the entry and tip coordinates (needle orientation). Voxels lying on this line were marked and considered to belong to the ablation needle. The temperature measurements to be considered are those along the B0 direction on each side of the marked needle-voxels. Three regions of interest (ROI) were marked, starting from the tip of the needle and working towards the entry. ROI 1 and 2 (the regions closest to the tip) are used to identify boiling/bubbles, while ROI 3 is used to identify when heating begins and ends. Each ROI had 56 voxels arranged as 2 sets (one for each side of the needle) of 4 lines along the B0 direction stacked on top of each other, each line being 7 voxels wide.

C. **Voxel classification (automatic)**: At each time step, classify the voxels according to the following keywords.

- "Heated": measured temperature variation is above 3°C.
- "Boiling": measured temperature variation is below -3°C.
- "Neutral": measured temperature variation is between -3°C and 3°C.

The keyword "Boiling" here means that the "Boiling voxel" registered a boiling-induced negative artifact.

Then, for each ROI, compute the following ratios at each time:

$$R_h = \frac{\text{Nº of Heated voxels}}{\text{Nº of voxels in ROI}}, R_b = \frac{\text{Nº of Boiling voxels}}{\text{Nº of voxels in ROI}}, R_n = \frac{\text{Nº of Neutral voxels}}{\text{Nº of voxels in ROI}}.$$

A 1D median filter was applied to smooth each ratio in time.

Figure S13C exemplifies, for each ROI, pairs (to each side) of lines that contribute to the ratios. Figure S14 shows the behavior in time of the ratios for each ROI, in two representative cases.

D. **Event Detection (automatic):** At each time step, the ratios are checked to mark events according to the following.

- If {in ROI 3 $R_h > 0.2$} is true, then heating was considered on (event "HEAT ON"). Else, heating was considered off (event "HEAT OFF").
- When "HEAT ON" is true, if {in ROI 1 $R_b > R_h$ AND $R_b > 0.2$} is true, OR if {in ROI 2 $R_b > R_h$ AND $R_b > 0.2$} is true, then boiling is considered to occur (event "HEAT ON & BOILING"). Additionally, this also marks if boiling is occurring in ROI 1, or ROI 2, or both.

Additionally, heating was considered off (event "HEAT OFF") if more time has passed than the duration of the ablation since the beginning of "HEAT ON".

Because the heating device of this study is a monopole type microwave antenna, the radiating element is located at the tip, therefore the heating occurs more strongly in ROI 1 and ROI 2 than in ROI 3.

For the dataset of this study, the detection algorithm had an 80% rate of success (16 out of 20 cases): it successfully detected the appearance of boiling in 10 out of the 11 cases that had boiling-induced artifacts, and it successfully identified the absence of boiling in 6 out 9 cases that did not have the boiling-induce artifact. Representative examples are shown in Figure S14. The single false negative is due to the presence of a small boiling artifact. The three false positives were linked to the presence of significant residual movement which created transient temperature artifacts (due to the movement modifying the susceptibility).

In the cases where the on-the-fly detection algorithm was successful, it coincided with our manual detection of the boiling-induced artifact that was employed in the main text via observation of the negative temperature voxels (Figure S1). We have carried out this additional investigation as proof-of-concept that such detection may be automated. There are many aspects that may be improved and explored in future works dedicated specifically to detection of this artifact:

1. Besides the central slice, adjacent slices could also be considered, making the 3 regions of interest 3-dimensional (multi-slice).

2. The markings of the entry and tip points of the ablation needle, as well as its orientation, could be determined from the last images used at the time of needle placement, e.g. using real-time interactive images or T1/T2-

weighted images taken just before ablation. An even more automated option could be to train a Neural Network for pixel classification.

3. The use of the direct PRFS temperature measurements was made for the sake of simplifying this preliminary investigation, but more involved metrics could have been used: e.g., a running difference of the temperature images, or the difference of sliding window averages for “before” and “during” states.”

Supplementary Tables

Swine (B0) Abl. #	Dice unc. (%)	Dice corr. (%)	T.O. unc. (%)	T.O. corr. (%)	FNR unc. (%)	FNR corr. (%)
Pig 1 (1.5 T) Abl. 2	72	75	70	76	30	24
Pig 1 (1.5 T) Abl. 3	75	75	79	87	21	13
Pig 1 (1.5 T) Abl. 4	59	61	45	46	55	54
Pig 2 (1.5 T) Abl. 3	61	60	52	51	48	49
Pig 3 (1.5 T) Abl. 1	61	62	48	50	52	50
Pig 3 (1.5 T) Abl. 3	48	50	33	34	67	66
Pig 4 (1.5 T) Abl. 1	62	65	81	83	19	17
Pig 4 (1.5 T) Abl. 2	63	62	84	86	16	14
Pig 5 (3.0 T) Abl. 3	69	72	65	69	35	31
Pig 6 (3.0 T) Abl. 1	60	66	46	55	54	45
Pig 7 (3.0 T) Abl. 2	65	65	83	86	17	14
Mean	63	65	62	66	38	34
Median	62	65	65	69	35	31
IQR	7	8	33	34	33	34

Table S1. Summary of volumetric correlation measures for in-vivo preclinical experiments that had boiling artifacts. The 11 ablations that had boiling artifacts are identified in the first column with the swine and ablation numbers (referenced throughout the text), along with the respective B0 magnitude. Volumetric Dice coefficients, total overlap (T.O.) and false negative rates (FNR) are displayed for comparisons between thermometry data and T1w postprocedural data: “unc.” refers to uncorrected-and-T1w; “cor.” refers to corrected-and-T1w. Mean, median and interquartile range (IQR) are presented in the last three lines.

Swine (B0) Abl. #	Vol. T1w	Vol. unc.	Vol. cor.	Z T1w	Z unc.	Z cor.	Y T1w	Y unc.	Y cor.	X T1w	X unc.	X cor.
Pig 1 (1.5 T) Abl. 2	5.9	6.2	5.8	33.1	38.7	39.7	13.3	16.8	14.5	11.9	9.5	10.0
Pig 1 (1.5 T) Abl. 3	8.5	7.7	6.5	37.9	40.1	39.2	14.7	15.3	13.2	13.1	11.5	11.9
Pig 1 (1.5 T) Abl. 4	5.2	10.2	10.0	30.7	35.7	34.9	12.9	19.4	19.3	12.4	16.0	16.1
Pig 2 (1.5 T) Abl. 3	3.2	4.4	4.6	35.6	40.2	39.8	10.4	13.8	13.8	8.7	10.3	10.4
Pig 3 (1.5 T) Abl. 1	3.4	5.7	5.5	37.9	39.4	39.4	9.9	16.0	16.1	9.5	9.6	9.5
Pig 3 (1.5 T) Abl. 3	2.9	8.0	7.9	29.8	34.5	33.5	9.8	21.7	21.5	8.9	10.8	11.0
Pig 4 (1.5 T) Abl. 1	7.1	4.5	4.5	35.5	36.2	35.9	15.9	14.8	14.8	12.2	9.8	9.7
Pig 4 (1.5 T) Abl. 2	6.9	4.1	3.9	36.1	39.1	37.6	13.4	13.7	12.3	13.0	10.1	10.1
Pig 5 (3.0 T) Abl. 3	7.3	8.3	7.9	35.6	34.9	33.6	14.4	19.2	18.8	12.8	12.3	12.5
Pig 6 (3.0 T) Abl. 1	5.3	10.2	8.1	39.6	49.2	49.9	13.2	18.6	15.2	11.4	11.9	11.8
Pig 7 (3.0 T) Abl. 2	6.4	4.1	3.9	42.2	41.4	40.1	14.3	11.0	10.7	10.8	10.0	10.1
Mean	5.7	6.7	6.2	35.8	39.0	38.5	12.9	16.4	15.5	11.3	11.1	11.2
Median	5.9	6.2	5.8	35.6	39.1	39.2	13.3	16.0	14.8	11.9	10.3	10.4
IQR	2.7	3.8	3.4	3.6	4.2	4.4	2.7	4.6	3.9	2.5	1.8	1.8

Table S2. Summary of geometric measures for in-vivo preclinical experiments that had boiling artifacts. The 11 ablations that had boiling artifacts are identified in the first column with the swine and ablation numbers (referenced throughout the text), along with the respective B0 magnitude. Geometric measures are displayed for the lesion of each experiment based on: ("unc.") thermal dosage model for the original thermometry data; ("cor.") thermal dosage model for the corrected thermometry data; (T1w) T1w postprocedural data. "Vol." refers to the volume of the lesion in cm^3; "Z", "Y" and "X" refer to the length of the three main ellipsoidal axes in mm. Mean, median and interquartile range (IQR) are presented in the last three lines.

Supplementary Figures

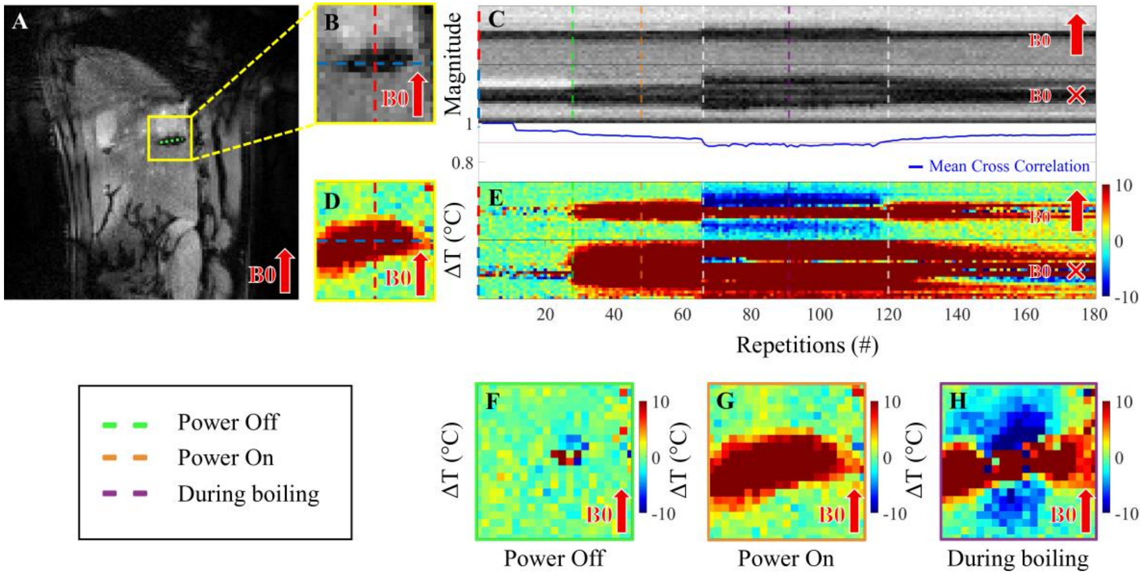

Figure S1. Representative case at 1.5T (pig 1, abl. 3) of susceptibility artifacts through time during liver MWA in an in-vivo porcine model. The left panel (A) displays a sagittal image. Zoom views of the magnitude (B) and temperature (D) images are shown at the vicinity of the MW probe's tip (approximately marked by a dotted green line). The magnitude (C) and temperature (E) profiles in head-foot (red dashed line) and anterior-posterior (blue dashed line) direction are plotted as a function of dynamic acquisition, together with the intercorrelation coefficient computed for each dynamic between the current frame and the reference frame (beginning, before ablation). Temperature maps are plotted before the ablation (F, green dashed line dynamic), during the ablation without boiling (G, orange dashed line dynamic) and with boiling (H, purple dashed line dynamic). The artifact occurs in the dynamics between the white dotted lines. Notice the relative stability of the artifact between transient periods: the intercorrelation coefficient of the magnitude decreases through each stage of the ablation, especially when boiling starts, but it reaches a plateau during the boiling period, indicating the stability. The same can be seen qualitatively in the temperature profile.

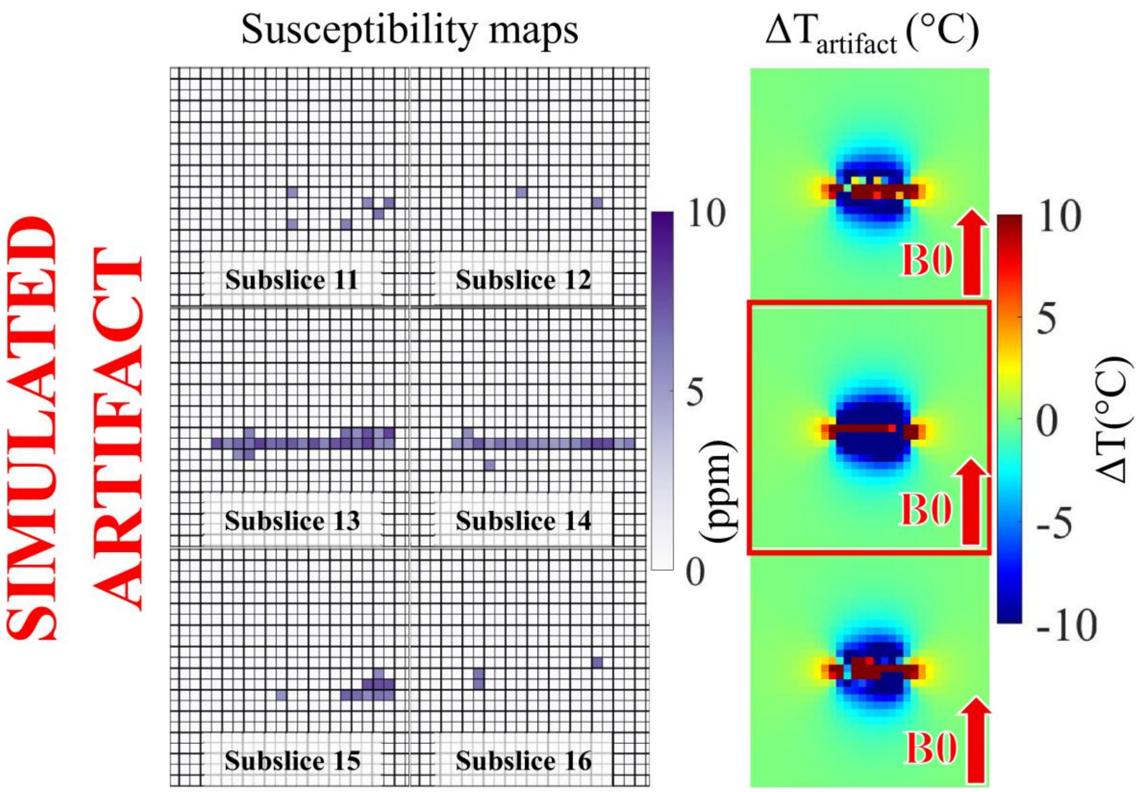

Figure S2. Numerical simulation: input data. Shown on the left panel, for each slice (each line), a random susceptibility distribution was set with a privileged location. The susceptibility distribution was set in a subvoxel grid generated by dividing the voxel resolution by 2 in every direction (thus, each slice corresponds to 2 subslices), and the simulated temperature artifact was regridded to native resolution (shown on the right panel, for each slice). Only the central slice (red border) will be used as input for the inverse problem to be solved by three different methods (see Figure 3 and text). The susceptibility depicted here is an introduced susceptibility change for the purposes of numerical simulation.

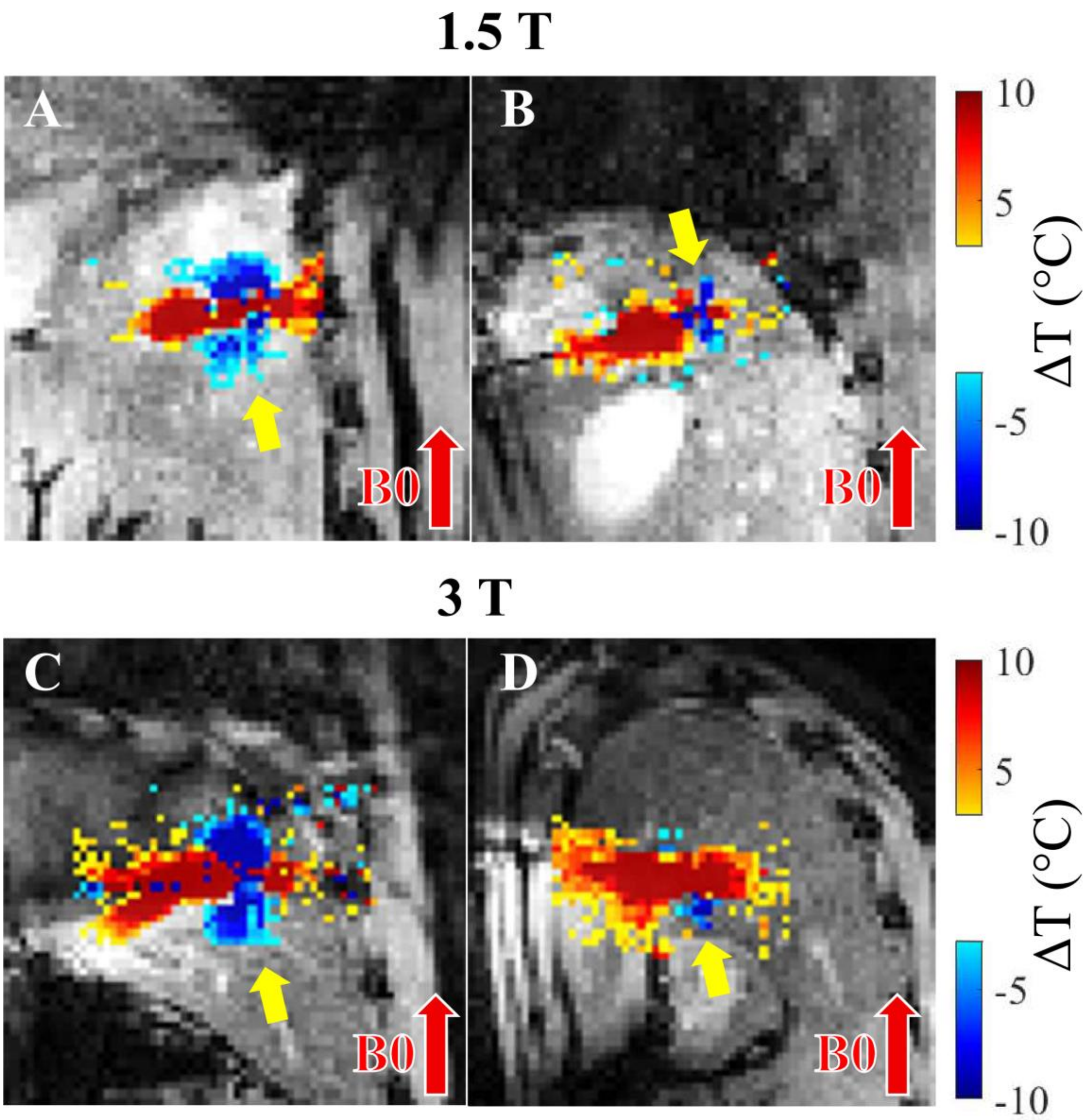

Figure S3. Representative cases of susceptibility artifacts during MWA in in-vivo swine. Temperature maps, overlaid on magnitude images with a cut-off between -2 and 2°C, showing susceptibility artifacts for MWA on in-vivo liver at B0 = 1.5T (top) and B0 = 3T (bottom). An approximate marking of the ablation probe position is given by a dotted green line. Artifacts are indicated by yellow arrows and are more clearly visible as unphysical negative temperature variations in blue. (A) pig 1, abl. 3; (B) pig 4, abl. 1; (C) pig 6, abl. 1; (D) pig 5, abl. 3. Although the general shape of the artifact is reminiscent of a dipole, and the B0 direction is the same in all images (bottom-top), these cases exemplify how the artifact appears on in-vivo data with very heterogeneous sizes, shapes and angular features.

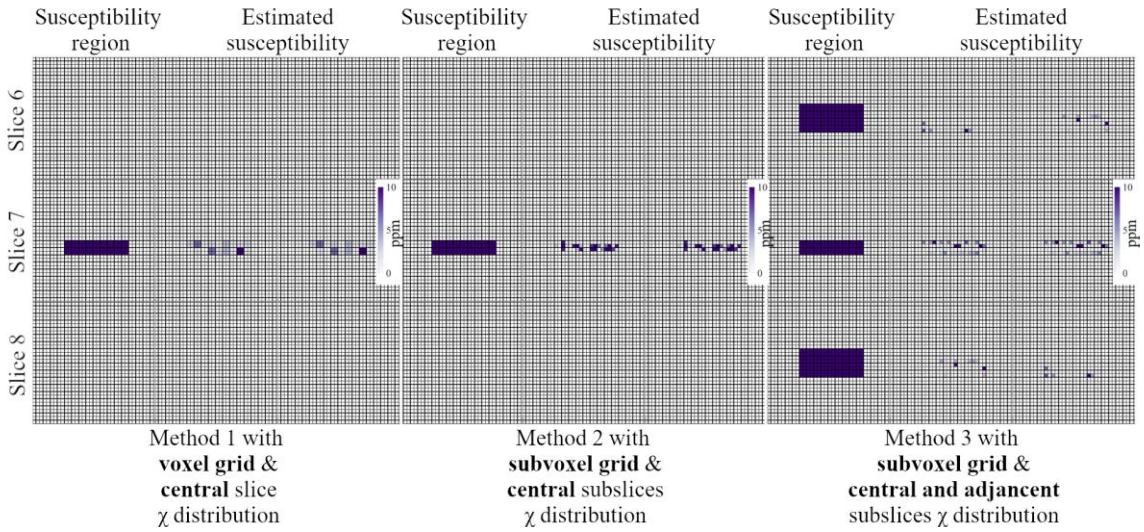

Figure S4. Numerical simulation: estimated susceptibility in three different methods. Left of each panel: the dark blue rectangles indicate the region where susceptibility values are going to be estimated and can possibly become non-zero. Middle and right of each panel: estimated susceptibility given by each method for each subslice. The subvoxel grid refers to dividing every direction of the voxel grid by 2 (thus, each slice corresponds to 2 subslices). Method 1 (left) uses only the central slice and a voxel grid. Method 2 (middle) uses only the central slice but with a subvoxel grid. Method 3 uses the central and adjacent slices with a subvoxel grid. The susceptibility distributions depicted here are estimated susceptibility changes for the purposes of numerical simulation.

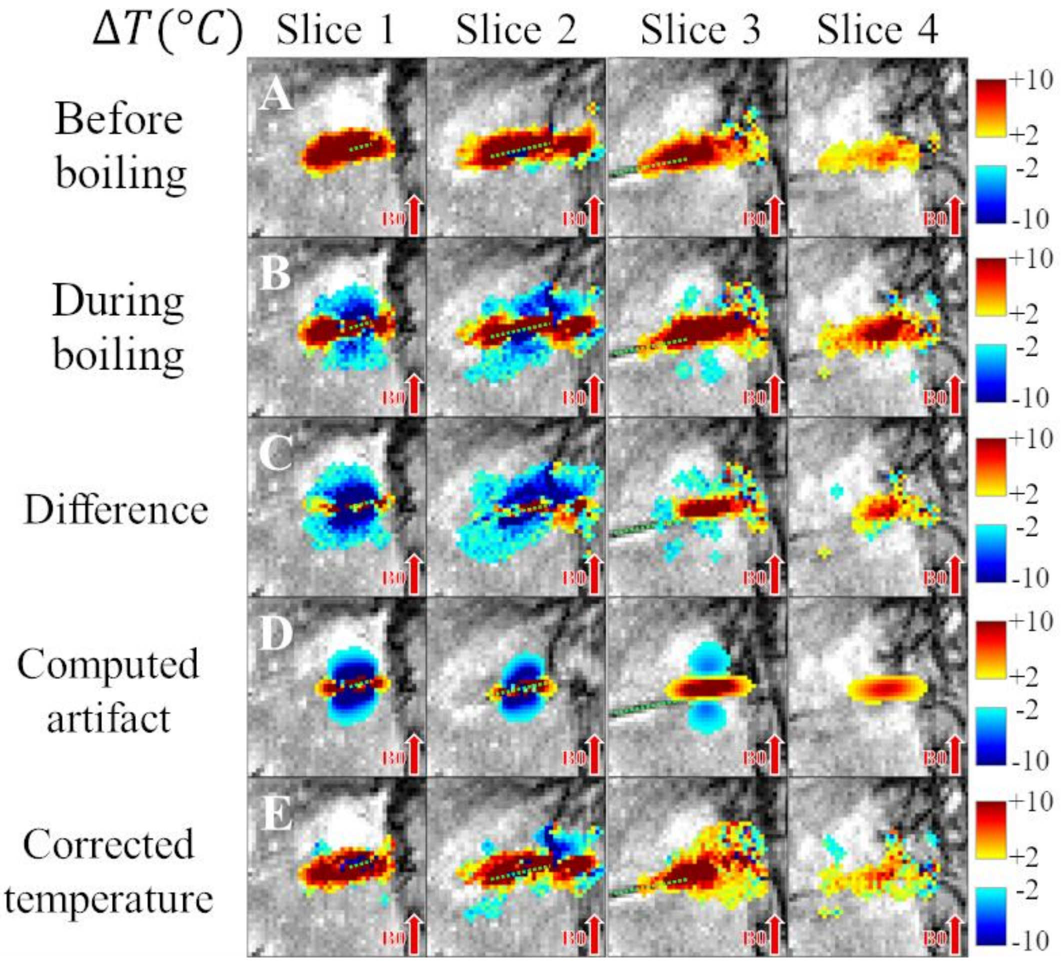

Figure S5. Spatial distribution of temperature in one of the in-vivo liver ablation experiments (pig 1, abl. 3) before and after correction. Temperature maps of four adjacent slices are overlaid on magnitude images with a cut-off between -2 and 2°C. An approximate marking of the ablation probe position is given by a dotted green line. For this particular case, the MW probe is going through the slices at an angle; the proximal end of the probe is seen in slice 3, goes through slice 2, and its tip (distal end) is in slice 1. Temperature maps before (A) and during (B) susceptibility artifacts. (C) A priori of corrupted temperature distribution related to the artifact appearance. (D) Estimated artifact. (E) Corrected temperature maps. The comments in the legend of Figures 4 and 5 apply.

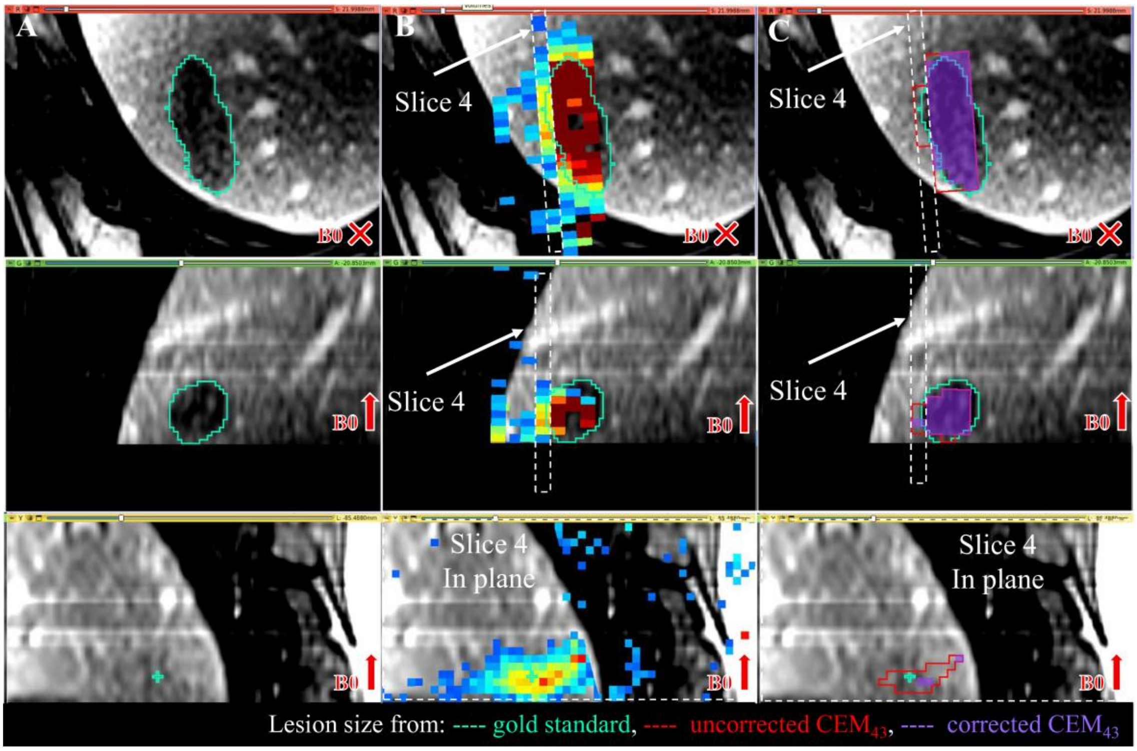

Figure S6. Comparison of TD versus T1w ablation size for slice 4 of experiment pig 1, Abl. 3. First column (A): Post-contrast T1w images and T1w ablation zones. Second column (B): Lesion size comparison from TD before (red) and after (purple) correction are overlaid on post-contrast T1w images. Third column (C): Comparison showing that post-ablative contrast-enhanced T1w agrees with the corrected TD estimation of classifying slice 4 as not ablated, which was predicted in Figures 6 and S9 after susceptibility correction.

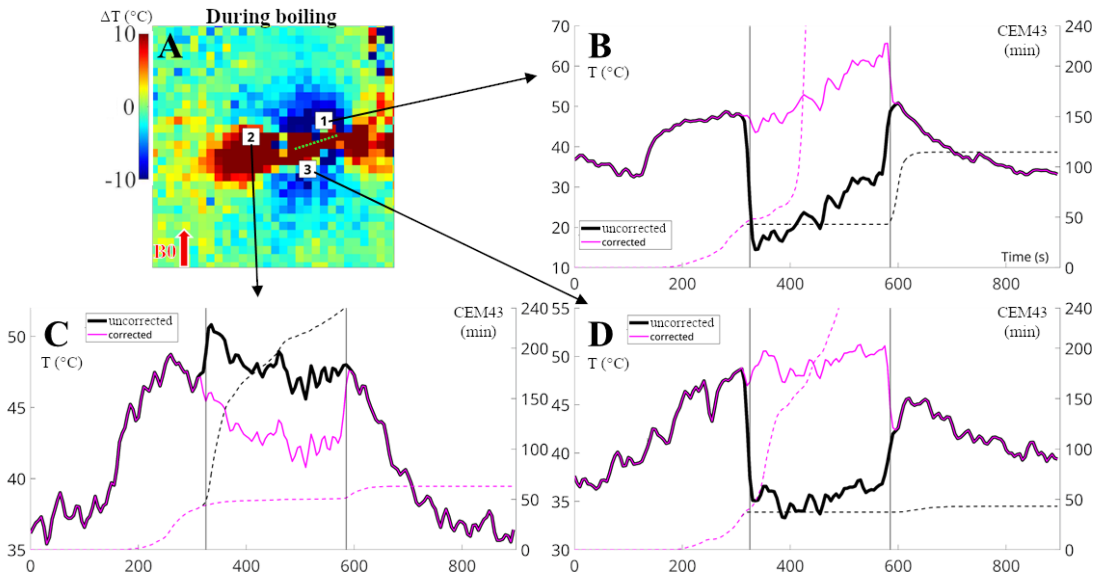

Figure S7. Temporal evolution of the temperature for slice 1 of in-vivo liver ablation experiment pig 1 Abl. 3, before and after correction. The temperature colormap shows temperature variation, while the time plots show the variation plus an offset of 37°C. (A) Temperature map during boiling-induced susceptibility artifact. (B,C,D) Temperature evolution (full lines) and CEM43 (dashed lines) are plotted for 3 voxels marked on (A). Vertical lines indicate when the correction starts and ends. An approximate marking for the position of ablation probe's tip is given by a dotted green line. The "bowtie" visual of the artifact is clear in space (A); and its effects can be seen in time as well: it causes overestimation of the temperature measurements in the positive lobes (C) and underestimation in the in negative lobes (B, D). After correction, the "temperature jumps" (up or down) are eliminated at the onset of boiling, and the time evolution returns to the general trend and scale seen before the appearance of the artifact. Notice that, after correction, the CEM43 estimation changes for each voxel: voxels 1 and 3 are now classified as ablated, while 2 no longer is. A jump of temperature is visible at the end of correction for some of the voxels (B, D), possibly due to the dipole artifact vanishing slowly due to gradual cooling. However, as this effect only occurs at the end of the ablation process, it does not affect the calculation of the thermal dose or the estimation of the lesion.

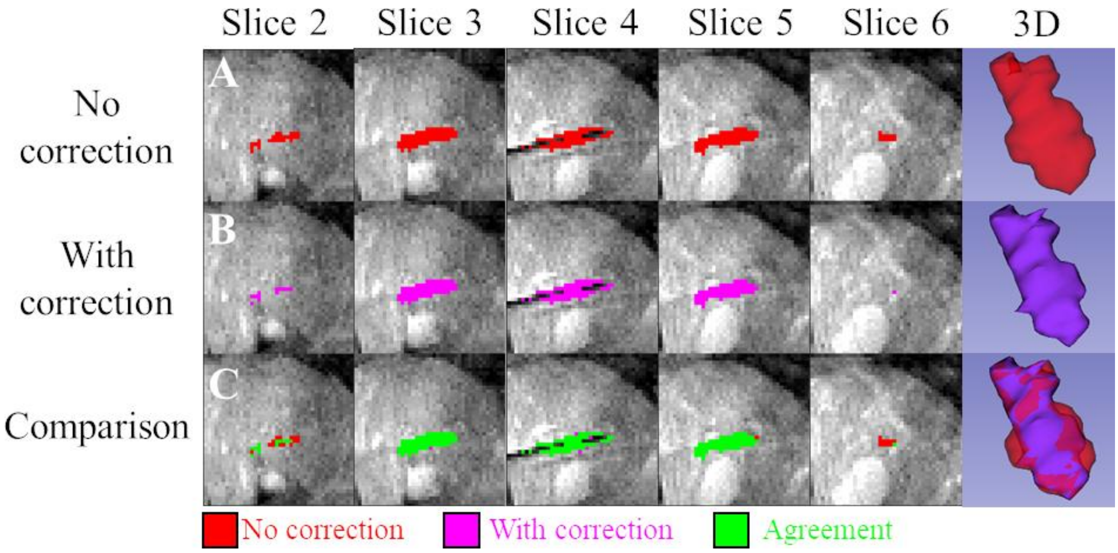

Figure S8. A case study (pig 1, abl. 2) for comparing the impact of the correction on dosimetry. In five consecutive slices, the estimated lesion size from CEM43 computation is overlaid over the magnitude images. A 3D representation of the lesion volume is displayed in the last column. The initial row (A) represents the uncorrected data set (red), the second row (B) depicts the proposed correction (purple), and the final row (C) illustrates the degree of agreement between the two data sets (green).

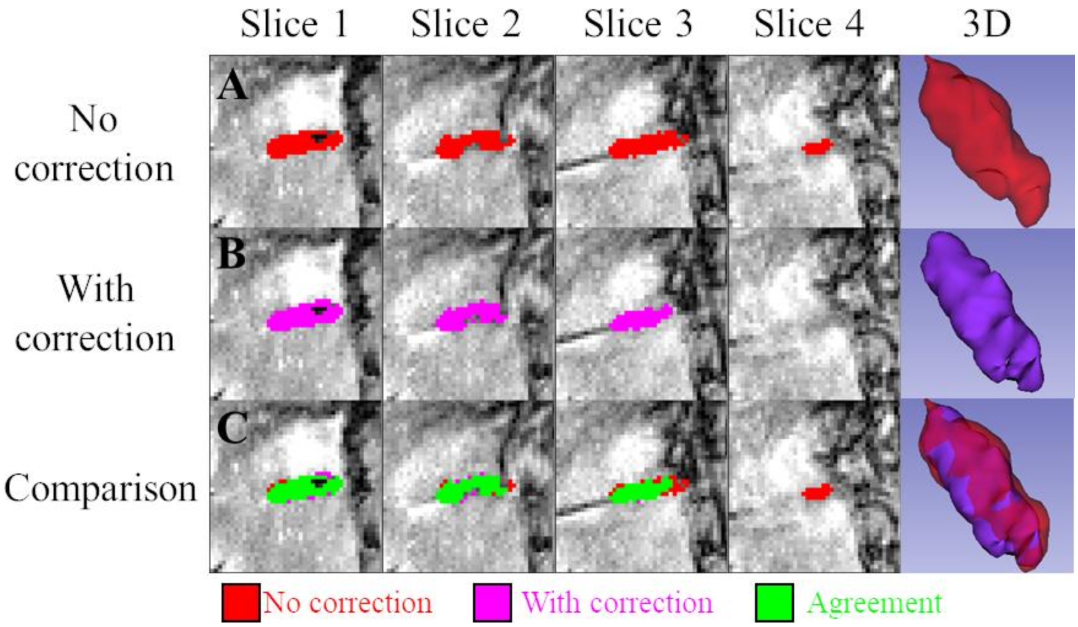

Figure S9. A case study (pig 1, abl. 3) for comparing the impact of the correction on dosimetry. In four consecutive slices, the estimated lesion size from CEM43 computation is overlaid over the magnitude images. A 3D representation of the lesion volume is displayed in the last column. The initial row (A) represents the uncorrected data set (red), the second row (B) depicts the proposed correction (purple), and the final row (C) illustrates the degree of agreement between the two methods (green).

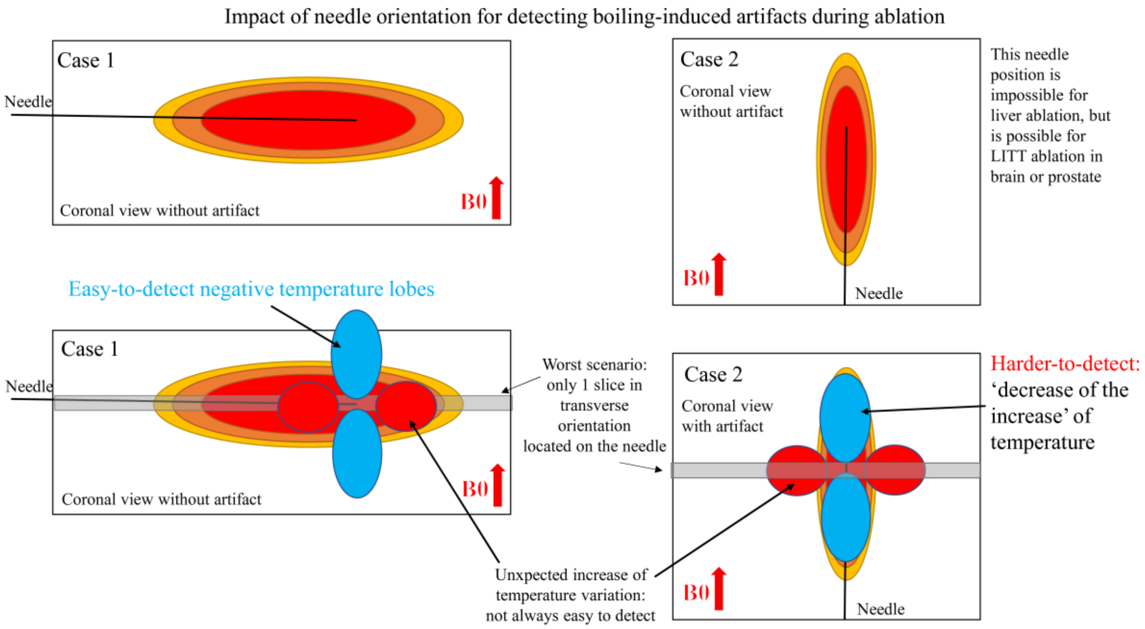

Figure S10. Examples of impact of needle orientation for detecting boiling-induce susceptibility artifacts. Coronal view of a hypothetical ablation with B0 Direction going bottom-top. Left: needle positioning perpendicular to the B0 Direction. Right: needle position parallel to B0 Direction. Depending on the orientation of the ablation needle with respect to B0, the negative and positive lobes of a boiling-induced dipole artifact may be harder to distinguish from the temperature distribution of the ablation, impairing the detection. The worst-case scenario occurs when only 2D imaging is being done and it is set along the positive lobes.

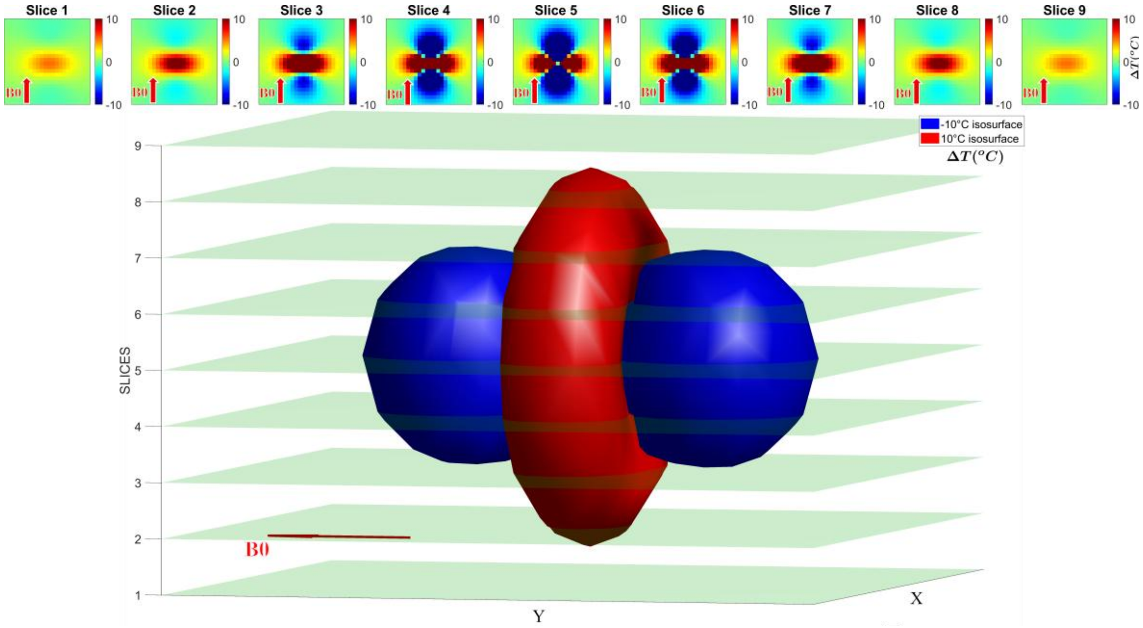

Figure S11. Simulation exemplifying the effect of multi-slice visualization over a dipole-shaped artifact. Nine slices acquired to visualize a dipole-shaped artifact generated by a susceptibility source positioned on slice 5. Top row: visualization of the artifact at each slice, 1 to 9. Bottom row: 3D visualization of the artifact with temperature isosurfaces of -10 and 10°C. Visualization of the classical shape of 2 positive lobes and 2 negative lobes will depend on which slice is being monitored and the temperature scale of interest. In slice 5, the 2D dipole shape is very clear. On slices 8 and 9, only the positive lobes are pronounced. This emphasizes the effect of an artifact on far slices. For this direction of B0 (bottom-top of slice image), the far-off slices will be mostly dominated by positive lobes. Multi-slice imaging is required for proper identification and correction of the 3D effect induced by the artifacts.

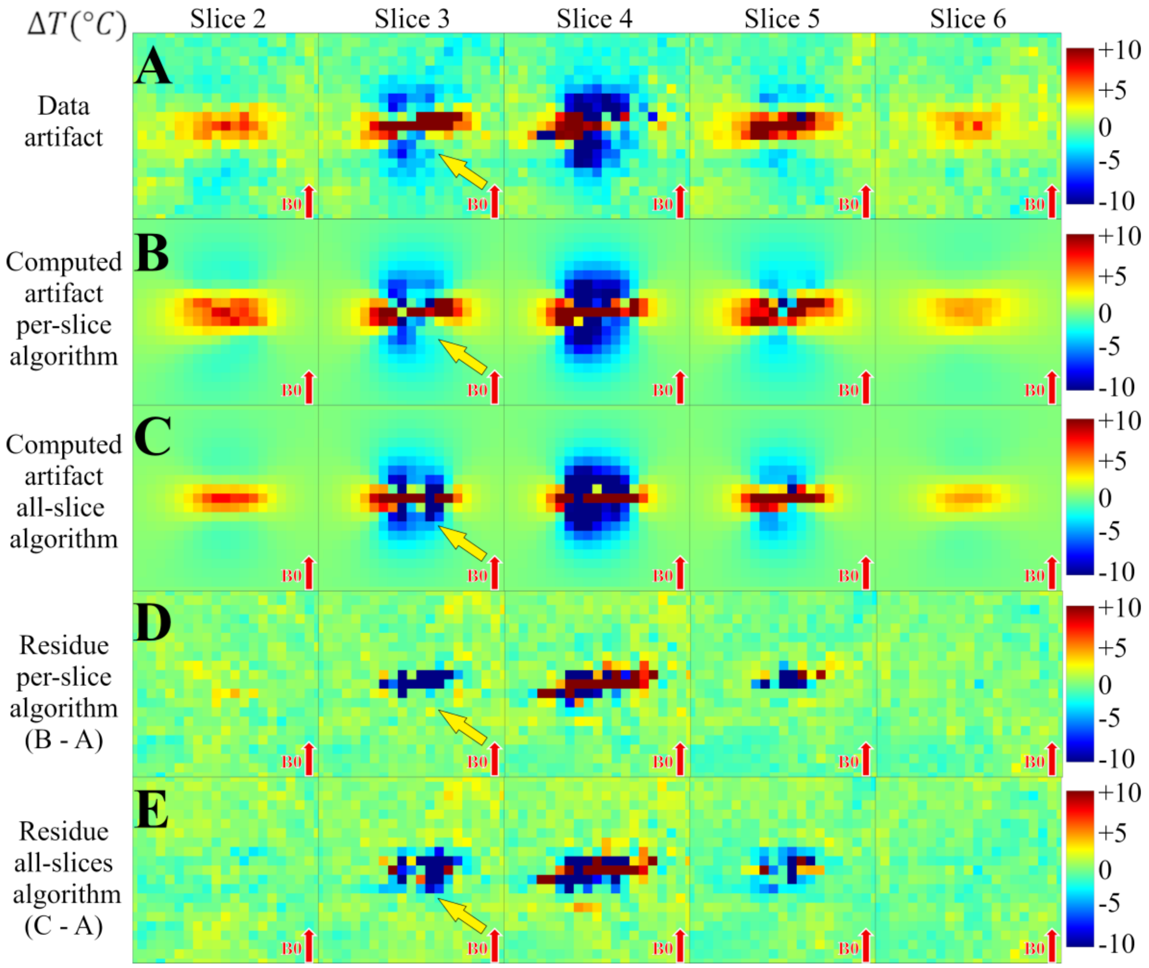

Figure S12. Comparison of the per-slice algorithm (2D correction) and all-slices algorithm (3D correction) in one of the in-vivo liver ablation experiments (pig 1, abl. 2). (A) A priori image of corrupted temperature distribution related to the artifact appearance. (B) Estimated artifact with the per-slice algorithm (algorithm used for the results in the main text). (C) Estimated artifact with the all-slices algorithm. (D) Residue for the per-slice algorithm (row B minus row A). (E) Residue for the all-slices algorithm (row C minus row A). For the per-slice algorithm, each 2D slice of data is used to generate a 3D susceptibility distribution which is used to correct that respective slice. In the all-slice algorithm, a single 3D susceptibility distribution is estimated based on all slices of data, which is used to correct all slices simultaneously. The all-slice algorithm gives similar results to the per-slice algorithm but is less reliable in terms of reproducing the intricate shapes of the boiling-induced artifact seen in data: the yellow arrow points to the major difference between the two approaches for this case.

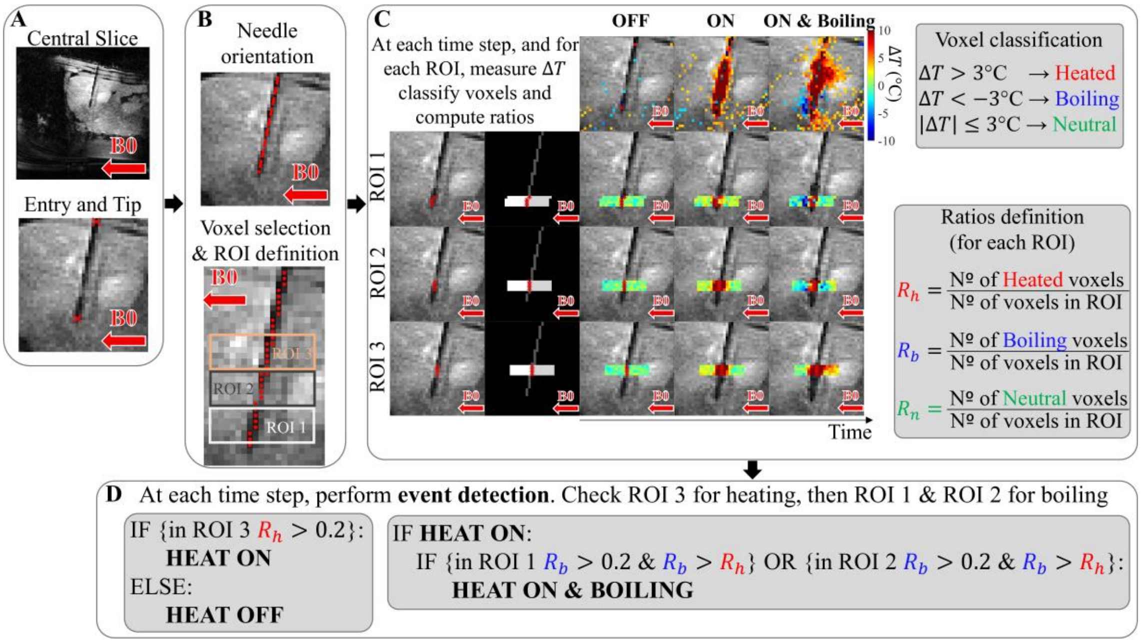

Figure S13. Flow chart of the image processing pipeline for the preliminary detection algorithm. (A) The central slice, as well as the end and entry point of the needle, are identified using two manually set markers (red crosses). (B) Voxels lying along the needle orientation (red line) are selected; 3 regions of interest (ROI) are identified. (C) At each time step, the temperature is used to classify the voxels as "Heated", "Boiling" or "Neutral", and for each ROI the ratios R_h, R_b and R_n are computed. (D) The ratios are used for event detection. Step (A) was performed manually before the ablation period. Steps (B), (C) and (D) are computed automatically. The keyword "Boiling" here means that the "Boiling voxel" registered a boiling-induced negative artifact. The presence of boiling is easily visible due to the negative temperature lobes (in blue). Temperature maps represent temperature variation, and the top temperature maps in (C) have a cut-off for low variation (-2 to 2°C). Temperature thresholds were applied for voxel classification; in particular, voxels with significant negative temperatures suggest boiling-induced artifact corruption. Arbitrary thresholds of 0.2 were fixed for R_h for deciding when heating is on and as a minimum of R_b for deciding when boiling occurs.

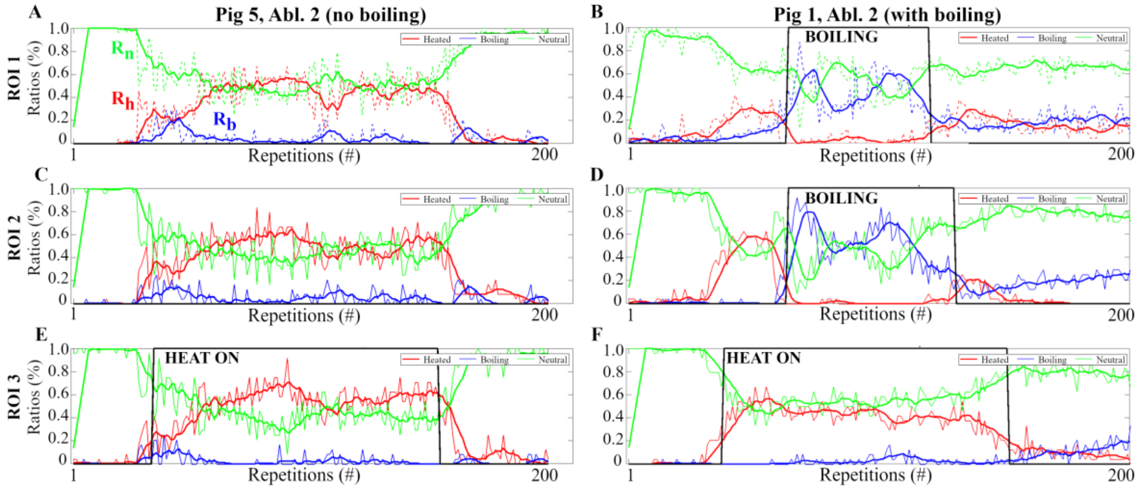

Figure S14: Representative cases of the detection algorithm in the presence (pig 1, abl. 2) and absence (pig 5, abl. 2) of the boiling-induced susceptibility artifacts. Panels (A, B), (C, D) and (E, F) show the ratios for the regions of interest (ROI) 1, 2, 3, respectively, which were defined for the detection algorithm (see Figure S13 and supplementary text). In each panel, the ratios R_h (Heated), R_b (Boiling) and R_n (Neutral) are displayed, respectively, in red, blue and green, with dotted lines being the computed ratio, and solid lines being the result of the use of the median filter in time. ROI 3 (bottom panels) is used to identify when heating begins and ends, which is indicated by the black line. ROI 1 and ROI 2 are used to identify the absence or the presence of boiling-induced susceptibility artifacts (black lines indicate presence of boiling). On the left, the ratio R_b was lower than R_h, therefore no boiling-induced artifact was detected. On the right, R_b clearly surpasses R_h on ROI 1 and ROI 2 during the heating period (ROI 3), therefore, the artifact was detected.

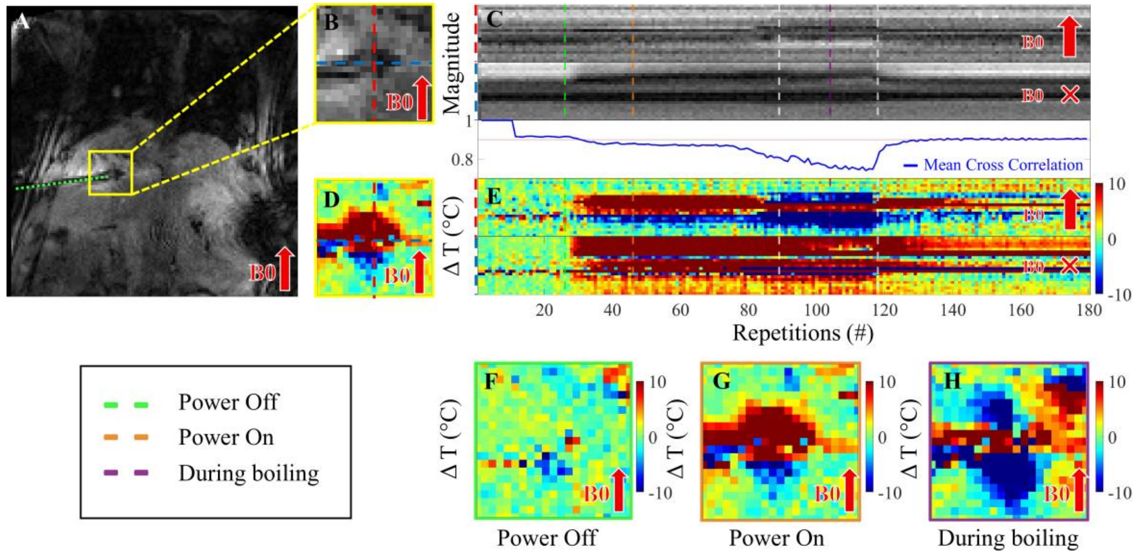

Figure S15. One case at 3T (pig 5, abl. 3) of susceptibility artifacts through time during the MWA procedure. The legend of Figure S1 applies. A small artifact is also visible at the start of MWA, before the occurrence of the bigger and most impactful boiling-induced artifact. The bigger artifact occurs in the dynamics between the white dotted lines. A relative stability of the artifact between transient periods can be seen in the correlation curve, albeit after a longer transient period. This situation exemplifies a more complex situation where a transient artifact is seen before the main one brought by boiling. This transient artifact may have been caused by blood flow or gradual bubble formation.
